# Spontaneous intersibling polymorphism in the development of dopaminergic neuroendocrine cells in sea urchin larvae: impacts on the expansion of marine benthic species

**DOI:** 10.3389/fnins.2024.1348999

**Published:** 2024-04-10

**Authors:** Alexandra L. Obukhova, Marina Yu. Khabarova, Marina N. Semenova, Viktor V. Starunov, Elena E. Voronezhskaya, Evgeny G. Ivashkin

**Affiliations:** ^1^Koltsov Institute of Developmental Biology, Russian Academy Sciences, Moscow, Russia; ^2^Department of Invertebrate Zoology, St-Petersburg State University, Saint Petersburg, Russia; ^3^Zoological Institute, Russian Academy Sciences, Saint Petersburg, Russia; ^4^Severtsov Institute of Ecology and Evolution, Russian Academy of Sciences, Moscow, Russia

**Keywords:** sea urchins, dopamine, serotonin, post-oral neurons, neuroendocrine system, phenotypic plasticity, larval development, larval swimming

## Abstract

**Introduction:**

The plasticity of the nervous system plays a crucial role in shaping adaptive neural circuits and corresponding animal behaviors. Understanding the mechanisms underlying neural plasticity during development and its implications for animal adaptation constitutes an intriguing area of research. Sea urchin larvae offer a fascinating subject for investigation due to their remarkable evolutionary and ecological diversity, as well as their diverse developmental forms and behavioral patterns.

**Materials and methods:**

We conducted immunochemical and histochemical analyses of serotonin-containing (5-HT-neurons) and dopamine-containing (DA-positive) neurons to study their developmental dynamics in two sea urchin species: *Mesocentrotus nudus* and *Paracentrotus lividus*. Our approach involved detailed visualization of 5-HT- and DA-positive neurons at gastrula-pluteus stages, coupled with behavioral assays to assess larval upward and downward swimming in the water column, with a focus on correlating cell numbers with larval swimming ability.

**Results:**

The study reveals a heterochronic polymorphism in the appearance of post-oral DA-positive neuroendocrine cells and confirms the stable differentiation pattern of apical 5-HT neurons in larvae of both species. Notably, larvae of the same age exhibit a two- to four-fold difference in DA neurons. An increased number of DA neurons and application of dopamine positively correlate with larval downward swimming, whereas 5-HT-neurons and serotonin application induce upward swimming. The ratio of 5-HT/DA neurons determines the stage-dependent vertical distribution of larvae within the water column. Consequently, larvae from the same generation with a higher number of DA-positive neurons tend to remain at the bottom compared to those with fewer DA-positive neurons.

**Discussion:**

The proportion of 5-HT and DA neurons within larvae of the same age underlies the different potentials of individuals for upward and downward swimming. A proposed model illustrates how coordination in humoral regulation, based on heterochrony in DA-positive neuroendocrine cell differentiation, influences larval behavior, mitigates competition between siblings, and ensures optimal population expansion. The study explores the evolutionary and ecological implications of these neuroendocrine adaptations in marine species.

## 1 Introduction

The process of animal development is intricately linked to adaptability, involving mechanisms that optimize reproduction while balancing population stability. Plasticity, or the ability to adapt to diverse conditions, plays a critical role at the embryonic development level (DeWitt et al., 1998). Exploring these phenomena often requires investigating physiological aspects that reveal hidden features.

One fascinating aspect of adaptability lies in the plasticity of integrative integrated systems within organisms, such as the nervous and endocrine systems (Leroith et al., 1986). These systems collaborate to ensure the overall well-being of the organism, with development representing a series of adjustments animals make to thrive in their environments. These adjustments involve intricate mechanisms, some of which may not be immediately obvious due to their connection to physiology.

The biphasic life cycle, crucial for various aquatic animals (Degnan and Degnan, 2006; Nielsen, 2009, 2012), involves a free-swimming larval phase and a benthic or sessile adult stage. Larval swimming relies on active migration in the planktonic environment, primarily driven by biogenic monoamines such as serotonin (5-HT) and dopamine (DA), orchestrating the coordinated beating of locomotory cilia (Conzelmann et al., 2011). The ciliary beating pattern, determined by the levels of these substances and the number of neurons producing them, varies across species with distinct developmental strategies, underscoring the intricacies of developmental adjustments.

Sea urchins, a classical subject for studying larval swimming patterns, ciliary beating, adaptational polymorphism, and the role of 5-HT and DA in these processes, offer valuable insights (Wada et al., 1997; Adams et al., 2011). During the planktonic phase, larvae exhibit behaviors crucial for survival, including food uptake, distribution, and horizontal migration, all linked to ciliary beating along larval arms. The regulation of ciliary activity is mediated by classical monoamines and other neurotransmitters. Moreover, DA plays a complex role in sea urchin larval development, influencing locomotion, responses to food, and metamorphosis (Burke, 1983; Wada et al., 1997; Adams et al., 2011). This integrative function of classical neurotransmitters, conserved across various animals, including vertebrates, makes it one of the most enduring neuroendocrine systems in Bilateria’s evolution (D’Aniello et al., 2020).

Both 5-HT and DA neurons play pivotal roles in the regulation of ciliary activity in specialized larval structures known as ciliary bands, which serve as the principal swimming and feeding organs of the larvae. Previous studies have demonstrated that the application of both monoamines stimulates cilia at the early post-hatching stage, while at late gastrula and pluteus stages, the effects tend to become opposite: stimulatory for 5-HT and suppressive for DA (Soliman, 1983; Mogami et al., 1992; Yaguchi and Katow, 2003). Correspondingly, 5-HT increases and DA decreases the beat frequency averaged over the ciliated epithelium, demonstrating stabilized and fluctuating effects on the phases of ciliary stroke (Wada et al., 1997; Shiba et al., 2002). Consequently, the application of various monoamines can initiate retardation or acceleration of swimming speed and modulate the swimming direction in free-swimming sea urchin larvae (Yaguchi and Katow, 2003). Similar effects of monoamines on cilia activity have been demonstrated for the larvae of different animals. External application of 5-HT activates cilia beating and accelerates embryonic rotation within the egg in gastropods (Uhler et al., 2000; Kuang and Goldberg, 2001; Kuang et al., 2002). In the gastropod *Lymnaea*, both 5-HT and DA increase the rate of embryonic rotation with similar potency (Voronezhskaya et al., 1999; Goldberg et al., 2011). In bryozoans, *Bugula* larvae, 5-HT and DA demonstrate opposite actions in the regulation of locomotory-dependent phototaxis (Pires and Woollacott, 1997). Both 5-HT and DA are involved in ciliary activity regulation in vertebrates (Walentek et al., 2014; Zhang et al., 2018).

The sea urchins *Mesocentrotus nudus* and *Paracentrotus lividus* are common species in the Sea of Japan and the Mediterranean Sea, respectively. Both species share similarities in their ecology, being covered with spines and moving slowly along the seabed. These animals rely on swimming planktonic larvae for distribution. After gamete spawning and external fertilization, the embryo hatches at the blastula stage, and the developing larva swims upward to the subsurface layer. During their development, the larvae undergo a transformation, acquiring several pairs of arms and initiating migration along the water column. This migration is facilitated by both the active beating of cilia in the arms and the influence of natural ocean currents that passively carry the larvae. A competent larva, responding to environmental signals, settles down and undergoes metamorphosis into a new benthic juvenile. Both *M. nudus* and *P. lividus* have well-investigated development and have served as objects in a number of developmental and ecological studies (Agatsuma, 2020; Boudouresque and Verlaque, 2020).

Our research employs a combination of morphological analysis and behavioral assays to explore the coordinated effects of the DA and 5-HT systems on larval swimming patterns at the gastrula at 24 hours post fertilization (hpf) and 28 hpf, and four-arm pluteus stages (36 hpf and, 40 hpf) of two sea urchin species, *M. nudus* and *P. lividus*. We have observed that in both species, the stable differentiation pattern of 5-HT neurons is accompanied by significant heterochrony in the appearance of DA-positive cells in larvae at the same developmental stage. These variations in the balance of 5-HT and DA cells underlie the different potentials of same-age larvae for upward and downward swimming along the water column and suggest decreased sibling competition. This intricate interplay of neurotransmitters in sea urchin larvae sheds light on their phenotypic plasticity and adaptability. This insight is valuable for understanding how animals adapt to their environments, with potential applications in marine ecology and developmental biology.

## 2 Results

### 2.1 Developmental features of DA and 5-HT neurons in *M. nudus* and *P. lividus* pre-feeding larvae

In this study, we employed the formaldehyde glutaraldehyde histochemical method (FaGlu) to visualize monoamines, specifically DA, in sea urchin larvae of *M. nudus* and *P. lividus*. This method allowed for efficient, rapid, and highly specific identification of DA-positive cells. Furthermore, it facilitated the detection of both DA-expressing cells in intact larvae and cells capable of uptaking DA (DA-uptaking) in larvae treated with a low concentration of DA. The majority of cells visualized using the FaGlu method exhibited angular morphology and emitted DA-positive processes toward each other. Our observations also suggest a correspondence between DA-uptaking cells in DA-treated larvae at early stages and DA-expressing cells in intact larvae at later developmental stages. The spatial distribution and morphological characteristics of all visualized DA-positive cells allowed for their identification as DA-positive neurons.

To visualize 5-HT-containing neurons (5-HT-neurons) in the larval apical organ, we employed immunoreactivity against 5-HT. This methodological approach facilitated quantitative analysis of both DA-expressing and DA-positive neurons, as well as 5-HT-neurons, throughout the larval pre-feeding stages of development.

**Figure 1:**
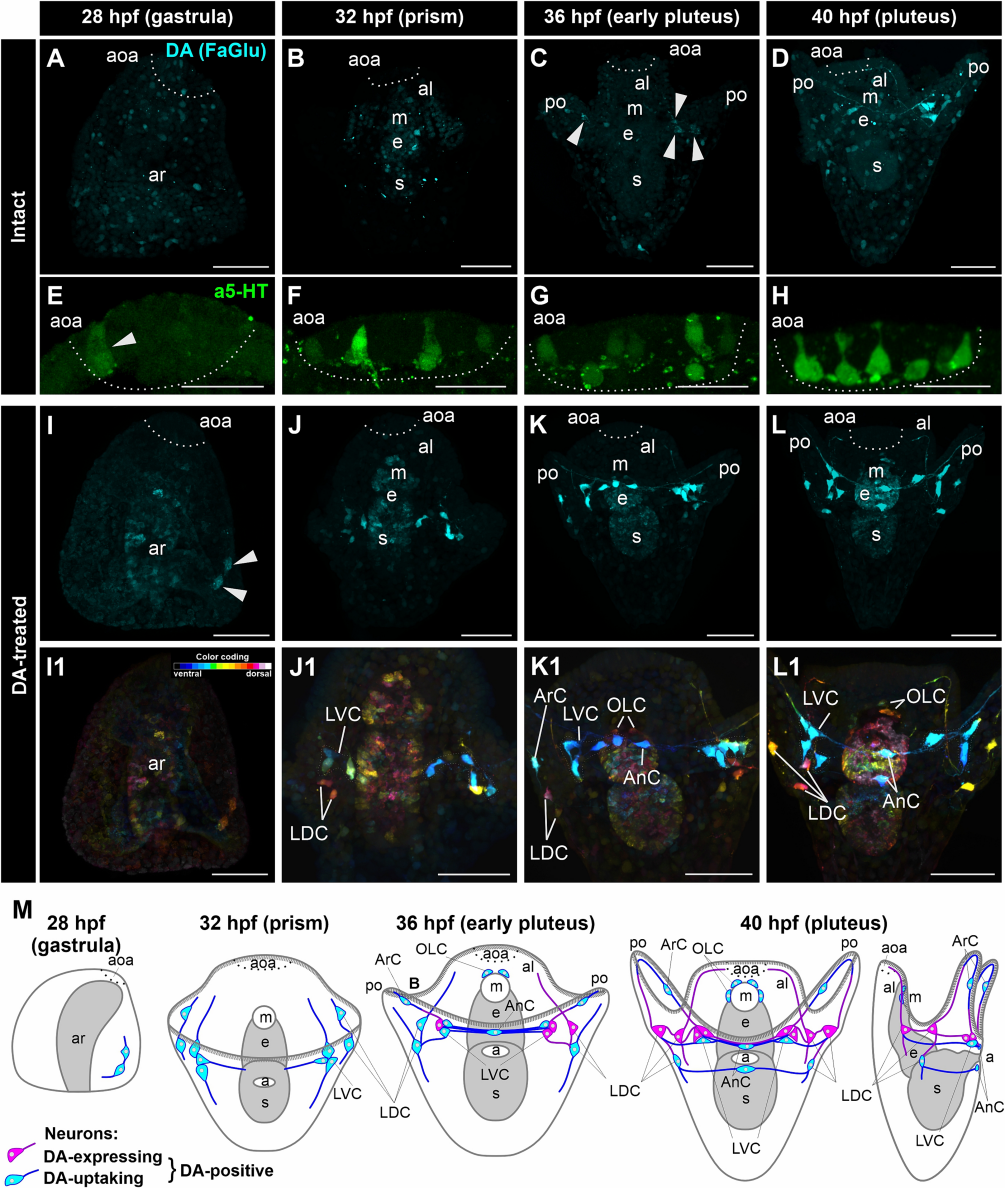
Developmental characteristics of DA-expressing, DA-positive, and apical 5-HT neurons in *M. nudus* larvae. **(A–D,I–L1)**, – Histochemical visualization (FaGlu, cyan) of DA-expressing neurons and **(E–H)** immunocytochemical detection (a5-HT, green) of 5-HT neurons in larvae spanning from the gastrula to the pluteus stages. **(A–D)** – Evolution and progressive augmentation of DA-expressing neurons from the gastrula to pluteus stages, with arrowheads denoting the initial four DA-expressing cells. **(E–H)** – Incremental rise from one to eight 5-HT neurons within the apical organ. **(I–L)** – Incremental augmentation from two at 28 hpf (marked by arrowheads) to 14 at 40 hpf of DA-positive neurons in larvae treated with DA. Notably, the majority of post-oral DA-positive neurons are linked with the arm edge. **(I1–L1)** – Color-coded representation of DA-positive neurons, delineating ventral (green) and dorsal (red) positions within the larval body. The lateral dorsal cells (LDC) and lateral ventral cells (LVC) within the post-oral arm region are situated on the dorsal and ventral sides, respectively. **(M)** – Schematic illustrations depicting DA-expressing (magenta) and DA-uptaking (cyan) neurons during the developmental progression of *M. nudus* larvae. The overall count of DA-positive neurons exhibits a steady increase, with the count of DA-uptaking neurons eventually surpassing that of DA-expressing neurons. AnC, unpaired cell near the anus; aoa, apical organ area; al, anterior lobe; ar, archenteron; ArC, arm cells; e, esophagus; LDC, lateral dorsal cells; LVC, lateral ventral cells; m, mouth; OLC, oral lobe cells; po, post-oral arms; s, stomach. Scale bars: **(A–D,E–L,E1–L1)** — 50 μm; **(E–H)** — 20 μm.

In intact *M. nudus* larvae, we did not observe DA-expressing cells at the gastrula and prism stages (28 and 32 hpf, respectively; Figures 1A,B). However, a group of DA-expressing cells emerged at the base of each post-oral arm during the early pluteus stage at 36 hpf (Figure 1C), persisting through the pluteus stage at 40 hpf (Figure 1D). Concurrently, 5-HT-neurons began to appear in the apical organ area at the gastrula stage (Figure 1E), gradually increasing in number during the transition from prism to pluteus (Figures 1F–H). Consequently, both the number of DA-expressing neurons in the post-oral region (Figure 1M) and 5-HT-neurons in the apical organ progressively increased during *M. nudus* larval development.

In DA-treated *M. nudus* larvae, a group of DA-uptaking cells was identified on the ventral side closer to the vegetal pole as early as the gastrula stage (28 hpf) (Figures 1I,I1). By the prism stage (32 hpf), cells condensed into a group at the base of the post-oral arms (Lateral Ventral Cells, LVC), while the bodies of two cells were positioned at the base of the oral lobe (Lateral Dorsal Cells, LDC group; Figures 1J,J1). During the early pluteus stage (36 hpf), solitary cells emerged on the post-oral arms (Arm Cells, ArC), with their processes extending near the arm edges. Notably, the left and right groups at the base of the post-oral arms were interconnected by several processes, along with an unpaired solitary cell located near the anus (Anal Cells, AnC; Figures 1K,K1). Additionally, two cells were situated near the mouth opening (Oral Lobe Cells, OLC, Figures 1K,K1). By the pluteus stage (40 hpf), the LDC group encompassed three cells, with an unpaired cell adjacent to the anus (AnC). Furthermore, a group of DA-uptaking cells was located near the mouth opening (OLC; Figures 1L,L1). The majority of cells exhibited triangular-shaped multipolar morphology, emitting processes toward each other thus demonstrating an unambiguous neuronal phenotype (Figures 1L,L1). The post-oral LVC and LDC neurons were interconnected by a bundle of processes, most of which traversed near the arm edge (Figures 1K–L1). Throughout all developmental stages, archenteron cells exhibited slight staining, indicating the possible ability of archenteron and forming stomach cells to uptake DA (Figures 1I–L,I1–L1).

Following incubation with DA, FaGlu-induced fluorescence was elevated in both DA-expressing and DA-uptaking neurons. Comparative morphological analysis of intact and DA-treated larvae allowed discrimination between DA-expressing neurons capable of synthesizing dopamine and DA-uptaking neurons possessing a specific DA transporter. The comparison of DA-positive neurons demonstrated a prevalence of DA-uptaking neurons over DA-expressing neurons at each larval stage (Figure 1M). The spatial distribution of cell bodies and their projections along the arm edge suggests an association of all DA-positive post-oral neurons with arm ciliary bands.

**Figure 2:**
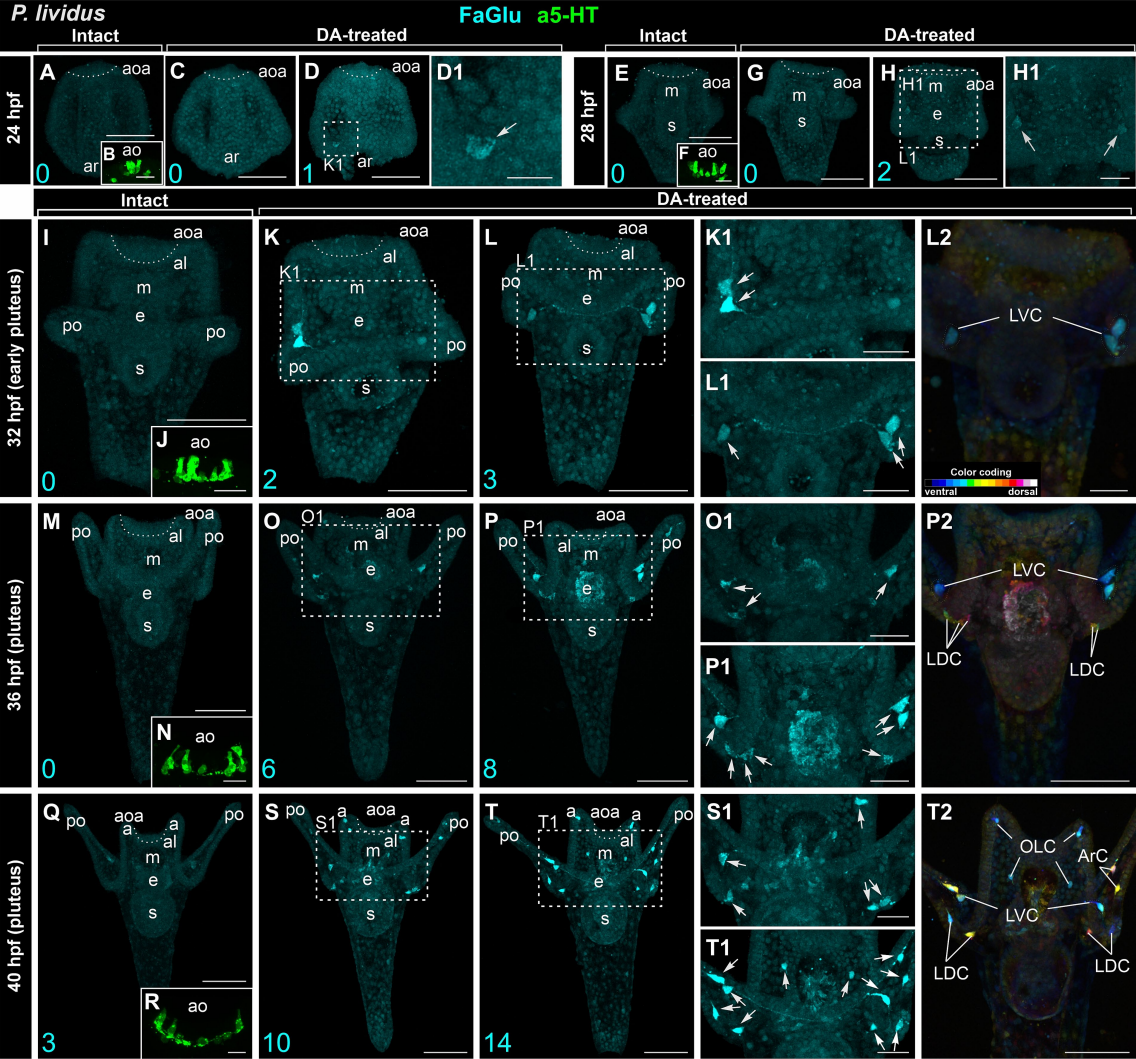
Developmental features of DA-expressing, DA-positive, and apical 5-HT neurons in *P. lividus* larvae. **(A,E,I,M,Q)** – Histochemical visualization (FaGlu, cyan) of DA-expressing neurons in intact larvae. **(B,F,J,N,R)** – Immunocytochemical detection (a5-HT, green) of apical 5-HT neurons. It is noteworthy to observe a gradual increase in the number of DA neurons in larvae from the gastrula to pluteus stages. **(C–D1,G–H1,K–L2,O–P2,S–T2)** – DA-positive neurons in dopamine-treated larvae. A gradual increase in the number of DA-positive neurons can be observed in DA-treated larvae from the gastrula to pluteus stages. **(D1,H1,K1,L1,O1,P1,S1,T1)** – Higher magnification highlighting the post-oral arm region with arrows denoting the DA-positive neurons. Most post-oral DA-positive neurons are located near the arm edge. Notably, asymmetry in the appearance of DA-positive neurons is observed in pluteus stages. **(L2,P2,T2)** – Color-coded depiction of DA-positive neurons, distinguishing from ventral (green) to dorsal (red) positions within the larval body. The lateral dorsal cells (LDC) and lateral ventral cells (LVC) within the post-oral arm region are situated on the dorsal and ventral sides, respectively. Cyan numbers in the bottom left corner represent DA-expressing or DA-positive neuron counts in the respective sample. al, anterior lobe; aoa, apical organ area; ar, archenteron; ArC, arm cells; e, esophagus; LDC, lateral dorsal cells; LVC, lateral ventral cells; m, mouth; OLC, oral lobe cells; po, post-oral arms; s, stomach. Scale bars: **(A–D,E–H,I,K–M,O–Q,P2,S,T,T2)** — 50 μm; **(K1,L1,L2,O1,P1,S1,T1)** — 20 μm; **(B,D1,F,H1,J,N,R)** — 10 μm.

In intact larvae of *P. lividus*, the pattern of appearance of DA-positive neurons and apical 5-HT neurons closely resembles that of *M. nudus*. At 24 hpf gastrula, no DA-expressing cells were found (Figure 2A), while six 5-HT neurons were located in the apical area (Figure 2B). DA treatment resulted in the appearance of a single DA-positive neuron in approximately 20% of the examined specimens (Figures 2C,D,D1). Intact 28 hpf gastrula possessed no DA-expressing cells (Figure 2E) and eight to 10 apical 5-HT neurons (Figure 2F). DA application allowed the revelation of two DA-positive neurons at the base of forming post-oral arms in most of the examined larvae (Figures 2G,H,H1). Intact 32 hpf and 36 hpf pluteus already had developed post-oral arms, however, possessed no DA-expressing neurons yet (Figures 2I,M), while the apical organ contained 10–12 5-HT neurons (Figures 2J,N). DA treatment resulted in the appearance of three DA-positive neurons at the base of post-oral arms at 32 hpf (Figures 2K–L2) and six to eight DA-positive neurons at 36 hpf larvae (Figures 2O–P2). Notably, marked asymmetry in DA-positive neuron differentiation was observed at the right and left sides of the larvae (Figures 2K1,L1,O1,P1). The first three DA-expressing neurons appeared only by 40 hpf pluteus (Figure 2Q) with concurrent 12 5-HT neurons constituting the apical organ (Figure 2R). In DA-treated larvae, the number of DA-positive neurons increased up to 10 and 14 (Figures 2S–T2).

The cell bodies primarily congregated at the base of post-oral arms (LDC and LVC in Figures 2S1–T2), with a solitary cell located along the arm edge (ArC in Figure 2T2). The DA-positive neurons of the LVC and LDC post-oral arm groups were connected by processes running along the arm (Figures 2T,T1). While in *M. nudus*, oral lobe neurons were preferentially concentrated around the mouth (Figures 1K1,L1,M), in *P. lividus*, only two OLC neurons were located close to the mouth, with another two situated at the edge of the anterior lobe (Figure 2T2). Notably, no DA-positive cell was observed in the anus region in *P. lividus*.

Despite their high similarity, *M. nudus* and *P. lividus* larvae exhibit specific differences in the timing and number of DA-positive neurons and 5-HT neurons appearance. Apical 5-HT neurons differentiate earlier than post-oral arm DA-positive neurons in both species. Additionally, DA-treated larvae in both species exhibit a prevalence of DA-positive neurons over DA-expressing neurons at each developmental stage. DA-positive neurons appear earlier in *M. nudus* than in *P. lividus* (28 hpf and 32 hpf, respectively), with the total number being lower in *P. lividus* by 40 hpf. The processes of post-oral DA-expressing and DA-positive neurons can be traced along the larval arm edge, indicating an association of these neurons with ciliary bands in the arms.

### 2.2 Heterochronic polymorphism in the appearance of DA neurons in sea urchin larvae

We observed a polymorphism in the presence of DA-positive neurons in DA-treated larvae of *P. lividus*, which was particularly pronounced at the early pluteus and pluteus stages (36 and 40 hpf). In larvae of the same age and from the same fertilization batch, the number of DA-positive neurons varied from six to eight at 36 hpf (Figures 2I–I1,N–N1) and from 10 to 14 at 40 hpf (Figures 2J,J1,O,O1).

**Figure 3:**
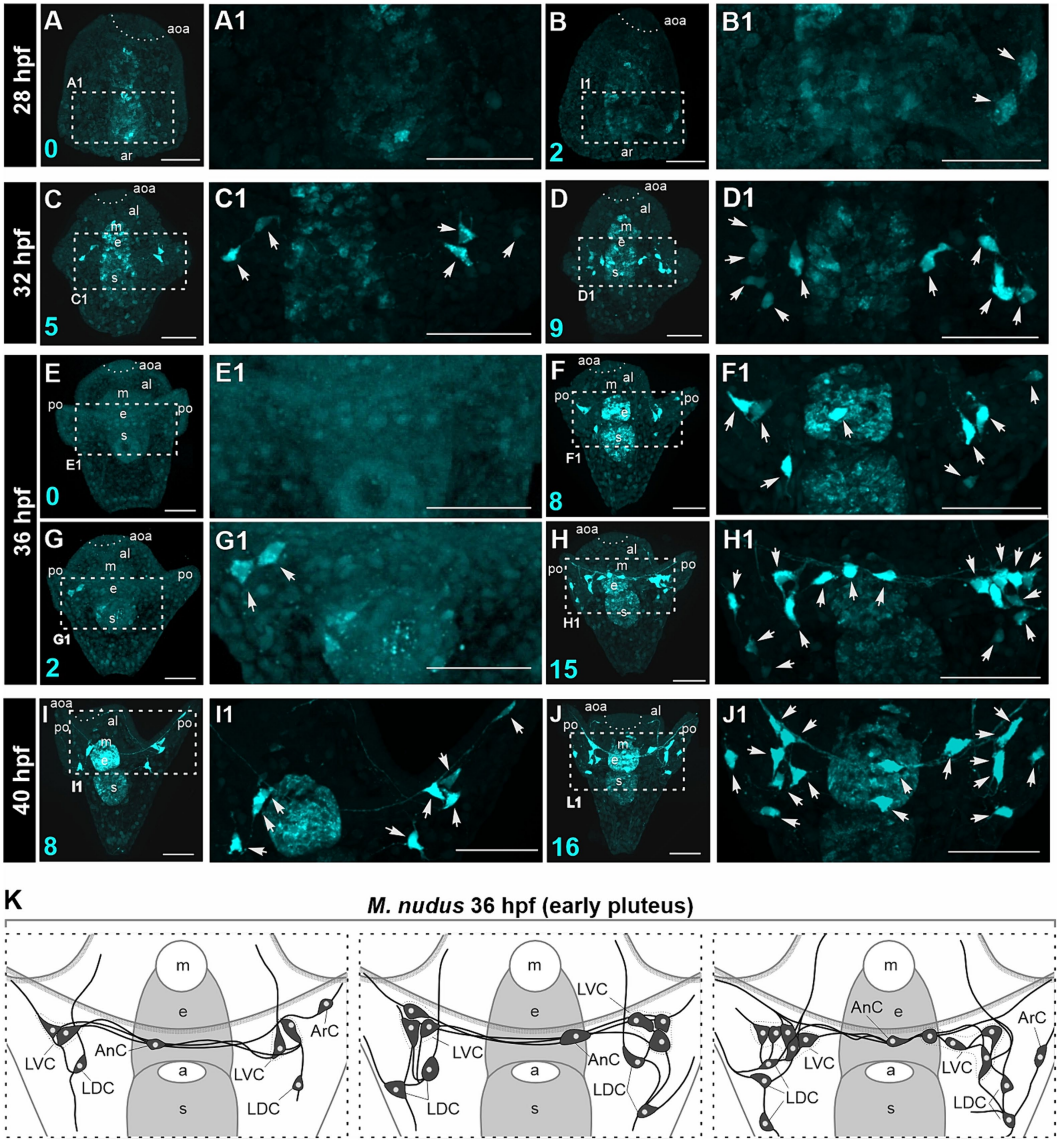
Polymorphism in DA-positive neuron number in larvae of *M. nudus*. DA-positive neurons were visualized using the FaGlu reaction (cyan) in representative larvae at subsequent developmental stages from 28 hpf gastrula to 40 hpf pluteus. **(A–J)** – General view and **(A1–J1)** high magnification of the DA-positive neurons. Arrowheads indicate the bodies of DA-positive cells. **(K)** – Schematic drawings of DA-positive neuron polymorphism at the early pluteus stage. Note the variation in the number of DA-positive neurons in the lateral dorsal cells (LDC) and lateral ventral cells (LVC) groups of post-oral arm cells. Cyan numbers in the left lower corner represent DA-expressing or DA-positive neuron counts in the respective larva. al, anterior lobe; aoa, apical organ area; ar, archenteron; ArC, arm cells; e, esophagus; LDC, lateral dorsal cells; LVC, lateral ventral cells; m, mouth; po, post-oral arms; s, stomach. Scale bars: **(A–J,A1–J1)** — 50 μm.

We conducted a detailed morphological examination of the polymorphism of DA-positive neurons using larvae of *M. nudus* ranging from 28 hpf gastrula to 40 hpf pluteus. For better visualization, all larvae were pre-treated with DA, thus ensuring that all visualized cells were DA-positive neurons (comprising both DA-expressing and DA-uptaking neurons). In *M. nudus* gastrulae, the majority of embryos lacked any DA-positive cells (Figures 3A,A1), while some individuals exhibited the presence of two DA-positive neurons (Figures 3B,B1). By the prism stage at 32 hpf, individuals with five to nine DA-positive neurons were observed (Figures 3C–D1). The greatest variability in DA-positive neurons was noted at the early pluteus stage at 36 hpf (Figures 3E–H1). While some individuals lacked or possessed only two DA-positive neurons (Figures 3E,E1,G,G1), others from the same fertilization group displayed counts of eight or even 15 DA-positive neurons (Figures 3F,F1,H,H1). At the pluteus stage at 40 hpf, all examined individuals demonstrated the presence of DA-positive neurons, with counts ranging from eight (Figures 3I,I1) to 16 (Figures 3J,J1). The majority of DA-positive neurons were associated with post-oral arms and the oral lobe, with cell processes extending underneath the arm edge (Figures 3H,H1,I–J1).

We examined larvae from different fertilizations to confirm the occurrence of heterochrony at the early pluteus stage at 36 hpf. We noted maximal variability in the LDC group located at the base of post-oral arms. This group typically consisted of three to five DA-positive neurons, while some individuals lacked these cells entirely. The LVC group at the base of the oral lobe exhibited variations in number from one to three, and AnC were present either as one or two unpaired neurons in different individuals. Furthermore, we noted asymmetry in the appearance and distribution of cells in the left and right groups. Heterochronic polymorphism in the presence of DA-positive neurons in each group is summarized in a diagram (Figure 3K).

**Figure 4:**
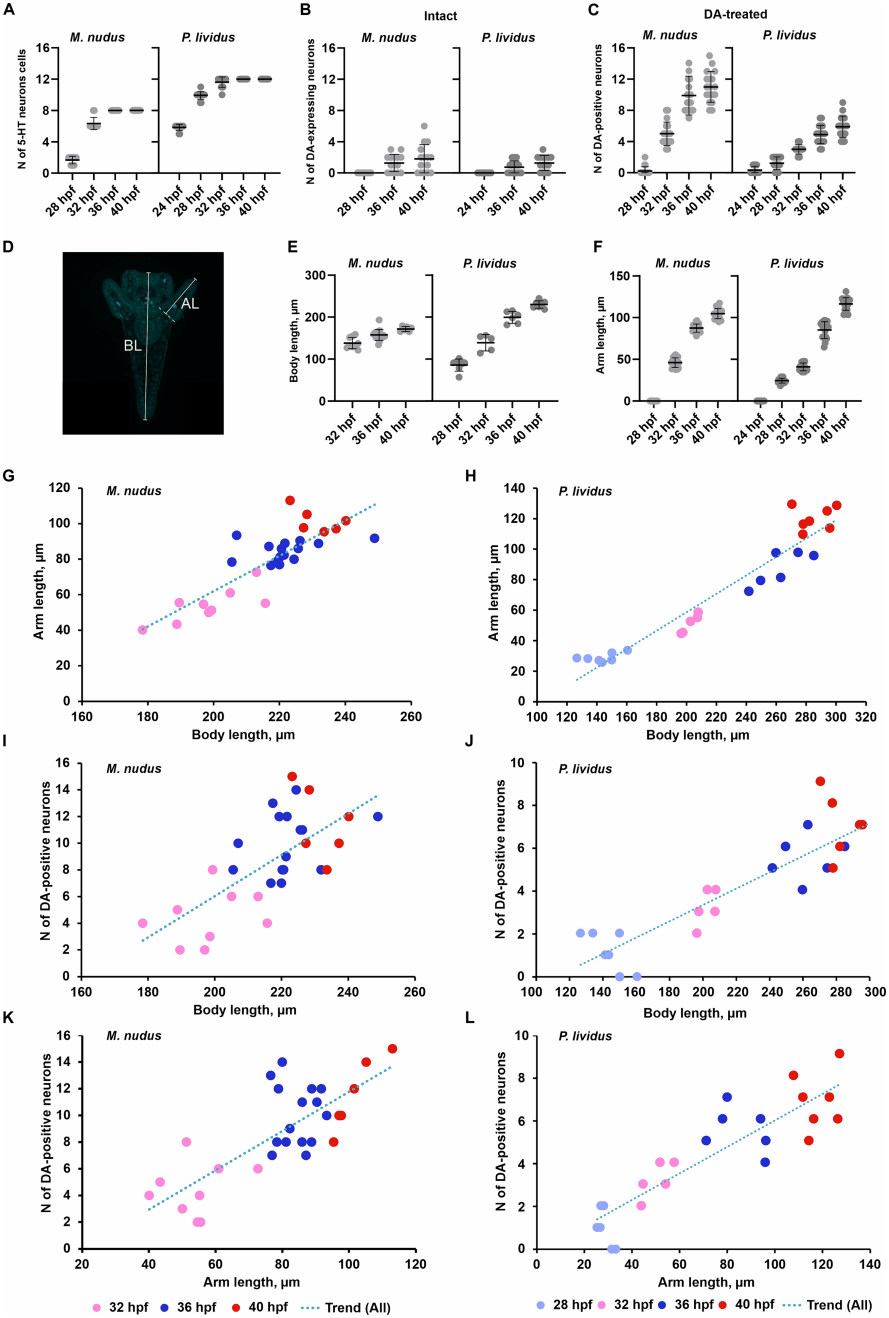
Number of neurons, arm and body lengths in pre-feeding larvae of *M. nudus* and *P. lividus*. **(A)** - Number of apical 5-HT neurons in larvae at different stages. It is noteworthy that there is minimal variability in cell numbers at each stage within both species. **(B,C)** - Number of DA-expressing neurons in intact larvae **(B)** and DA-positive neurons in DA-treated larvae **(C)** at different stages. There is significant variation in cell numbers within larvae of the same age, particularly notable for DA-positive neurons. **(D)** - Schematic representation of body length (BL) and post-oral arm length (AL) measurements. **(E,F)** - Body length **(E)** and arm length **(F)** of larvae at corresponding developmental stages. Mean ± SEM values are provided for graphs **(A–С)** and **(E–F)**. **(G–L)** - Linear regression graphs depicting the respective parameters during the *M. nudusp. Lividus*development of *M. nudus* **(G–K)** and *P. lividus* **(H–L)** larvae. Relationships between BL from gastrula to pluteus. **(I,J)** – Correlation between body length AL **(G,H)**, BL **(K,L)**, and post-oral arm length and the number of DA-positive neurons **(I,J)**, and AL and the number of DA-positive neurons are plotted. While the overall number of DA-positive neurons increases with age, significant variation exists among larvae of the same age. It is notable that larvae with longer arms and bodies may exhibit a smaller number of DA-positive neurons, whereas those with shorter arms and bodies may possess a larger number of DA-positive neurons. This variability is consistent across all examined developmental stages in both species.

Calculation of cells in larvae of different ages for both species corroborated our observations based on immunochemical staining of apical 5-HT-neurons and histochemical visualization of DA-positive neurons. While the number of 5-HT-neurons remained stable at certain larval stages (Figure 4A), DA-expressing neurons (in intact larvae) and DA-positive neurons (in DA-treated larvae) exhibited high variability among individuals of the same age (Figures 4B,C). In the case of DA-expressing neurons, this variation included individuals completely lacking DA-positive neurons even at 36 and 40 hpf pluteus (Figure 4B). For DA-positive neurons, larvae displayed twofold differences (e.g., 28 hpf) and even fourfold differences (36 hpf, 40 hpf) in counts (Figure 4C). Such significant differences in the number of DA-positive neurons in developing larvae were supported by a higher variance in their counts (Table 1). In contrast, the number of 5-HT-neurons demonstrated low variance (Table 1).

**Table 1:** Variances in the number of 5-HT- and DA-positive neurons, arm length and body length in larvae of *M. nudus* and *P. lividus* at subsequent stages of development.

*M. nudus*					
Variance	24 hpf	28 hpf	32 hpf	36 hpf	40 hpf
N of DA-positive neurons (DA-treated)	–	6.088	0.089	0.029	0.032
N of 5-HT neurons (Intact)	–	0.045	0.010	0	0
Arm length	–	–	0.017	0.003	0.004
Body length	–	–	0.007	0.007	0.001

*P. lividus*					
Variance	24 hpf	28 hpf	32 hpf	36 hpf	40 hpf
N of DA-positive neurons (DA-treated)	2.118	0.438	0.039	0.059	0.054
N of 5-HT neurons (Intact)	0.004	0.006	0.002	0	0
Arm length	–	0.015	0.014	0.015	0.004
Body length	–	0.032	0.024	0.005	0.001

To investigate whether differences in the number of DA-positive neurons correlated with larval age, we measured body length (BL) and post-oral arm length (AL; Figure 4D), which are characteristic features of larval developmental tempo. Both BL and AL parameters precisely reflected the larval age in both *M. nudus* and *P. lividus* (Figures 4E,F). Additionally, low variability in BL and AL was observed among larvae from the same generation (Table 2). AL and BL in selected individuals demonstrated a predicted linear positive age-related curve (Figures 4G,H). Similar linear positive correlations were observed for BL and the number of DA-positive neurons (Figures 4I,J) and AL and the number of DA-positive neurons (Figures 4K,L). However, there was no such correlation among representative larvae of the same age (see Spearman correlation coefficient, Table 2). Particularly, individuals with long arms and long bodies may have a smaller number of DA-positive neurons than larvae with short arms and short bodies (see examples in Figures 4I–L). Such discrepancies were found in all examined developmental stages of *M. nudus* and *P. lividus* (Figures 4I–L; Table 2).

**Table 2:** Spearman correlation coefficient for arm lengths and the number of DA-positive neurons in larvae of *M. nudus* and *P. lividus.*

*M. nudus*						
N of DA-positive neurons vs. Arm length	24 hpf	28 hpf	32 hpf	36 hpf	40 hpf	All stages
Correlation (Spearman)	–	0.201	0.242	0.095	0.123	**0.917**
*p*-value (Bonferroni corrected)	–	1.26	0.99	2.112	1.869	**<0,0001**

*P. lividus*						
N of DA-positive neurons vs. Arm length	24 hpf	28 hpf	32 hpf	36 hpf	40 hpf	All stages
Correlation (Spearman)	–	–	0.182	0.246	0.125	**0.879**
*p*-value (Bonferroni corrected)	–	–	1.404	0.975	1.86	**<0,0001**

Bold values indicates their statistical significance.

Thus, our data clearly demonstrate that sea urchin larvae exhibit significant polymorphism in DA-positive neuron differentiation. The appearance of both DA-expressing and DA-positive post-oral cells was variable among the larvae of the same age. The number of DA-positive neurons does not correlate with the body size or arm length within the larvae of the same age. To the contrary, the appearance of apical 5-HT-neurons is highly uniform and stage-dependent.

### 2.3 Impact of polymorphism in DA-positive neurons on larval swimming pattern

**Figure 5:**
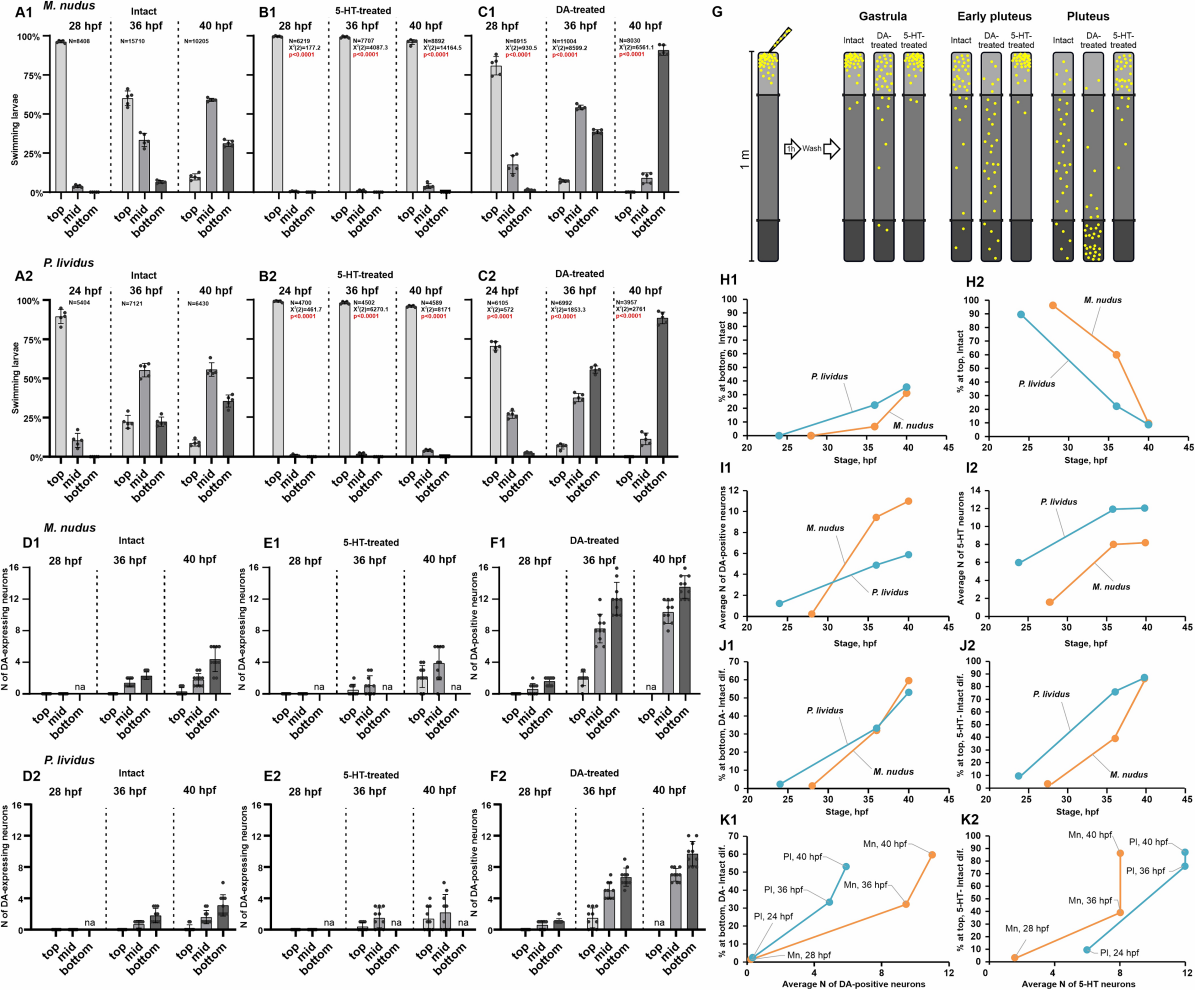
Larval swimming assays in relation to the number of DA-positive and 5-HT neurons. **(A1–C2)** – Spatial distribution patterns within a 1 m water column of intact **(A1,A2)**, 5-HT-treated **(B1,B2)**, and DA-treated **(C1,C2)** larvae of *M. nudus* and *P. lividus* at various developmental stages. Notable is the increasing larval abundance at mid and lower depths with developmental progression in intact and DA-treated cohorts. Conversely, 5-HT treatment consistently induces upward swimming across all developmental stages. ‘N’ denotes the total number of larvae assessed in all experimental runs. Each test was independently replicated five times, with a Chi-square test employed for comparison against the respective intact stage larvae results. Statistically significant differences were denoted by *p* < 0.0001. **(D1–E2)** – Quantification of DA-expressing neurons in representative larvae from top, middle, and bottom cohorts in intact and 5-HT-treated larvae of *M. nudus* and *P. lividus*. **(F1,F2)** – Quantification of DA-positive neurons in representative larvae from top, middle, and bottom cohorts in DA-treated larvae of *M. nudus* and *P. lividus*. It is noteworthy the increased presence of DA-expressing neurons in larvae from the bottom cohort in intact and 5-HT-treated groups across all stages, and DA-positive neurons predominantly in the middle and bottom cohorts in DA-treated larvae. Neuron counts were conducted in representative larvae from each tier at every examined developmental stage across all experimental groups. The number of larvae picked for neuron counting for each segment was 10–15. **(G)** – Schematic representation of the experimental setup and the results of the behavioral assay. **(H1–K2)** – Interspecies comparison of *M. nudus* (orange lines) and *P. lividus* (blue lines). **(H1,H2)** – Dependence of the percentage of larvae in the bottom **(H1)** and top **(H2)** layers on the larval developmental stage under intact conditions. **(I1,I2)** – Dependence of the average number of DA-positive neurons **(I1)** and 5-HT neurons **(I2)** on the larval developmental stage. **(J1,K1)** – Dependence of the magnitude of larval response to DA, expressed as the difference in the number of larvae at the bottom after DA treatment and in the control, on the developmental stage **(J1)** and the average number of DA-positive neurons at corresponding developmental stages **(K1)**. **(J2,K2)** – Dependence of the magnitude of larval response to 5-HT, expressed as the difference in the number of larvae in the surface layer after 5-HT treatment and in the control, on the developmental stage **(J1)** and the average number of 5-HT neurons at corresponding developmental stages **(K2)**.

DA and 5-HT are acknowledged regulators of ciliary beating and larval swimming in sea urchins. To probe the impact of heterochrony and varying quantities of DA-positive neurons at specific developmental stages on larval swimming behaviors, we conducted behavioral assays at three larval stages: 24–28 hpf (gastrula), 36 hpf (early pluteus), and 40 hpf (pluteus). The larvae were evaluated for swimming proficiency in 1-meter-long test tubes and stratified into three categories: upward swimming (top), intermediate (middle), and downward swimming (bottom). Additionally, we assessed the response of larvae at each stage to 5-HT (5-HT-treated) and DA (DA-treated) application (refer to the experimental scheme in Figure 5G). Concurrently, we quantified the number of DA-expressing neurons under normal conditions and in the 5-HT-treated groups, alongside DA-positive neurons in the DA-treated group.

In intact 24–28 hpf larvae, the majority demonstrated active upwelling (96.2% in *M. nudus*, and 89.5% in *P. lividus*), with only a small percentage in the middle (3.8 and 10.5%, respectively) and very few reaching the bottom (24 hpf, 28 hpf in Figures 5A1,A2). By 36 hpf, a notable proportion of larvae were in the middle (33.4% in *M. nudus*, and 55.3% in *P. lividus*) and bottom (6.6% in *M. nudus*, and 22.4% in *P. lividus*) groups. Consequently, 60.1% of *M. nudus* and 22.3% of *P. lividus* remained in upper layers (36 hpf in Figures 5A1,A2). By 40 hpf, the percentage of larvae in the middle and bottom groups increased in both species, reaching 59.0 and 31.2% in *M. nudus*, and 55.7 and 35.6% in *P. lividus*, with only 9.7 and 8.8% (in *M. nudus* and *P. lividus*, respectively) remaining at the top (40 hpf in Figures 5A1,A2). Thus, in untreated larvae, the proportion of the bottom group increased with age, while that of the top group decreased in both species.

Treatment with 5-HT altered the normal swimming pattern, with larvae of both species at all stages exhibiting strong ascending swimming, and only rare individuals remaining in the middle (maximum 3.9 and 4.0% at 40 hpf of *M. nudus* and *P. lividus*, respectively). No larvae of any age were found in the near bottom section (Figures 5B1,B2).

Preincubation with DA significantly altered the proportion of larvae located in the bottom group compared to intact larvae and contrasted with larvae incubated with 5-HT. At 24–28 hpf, only 80.9 and 70.8% of *M. nudus* and *P. lividus* were located at the top, while 17.7 and 1.4% were situated in the middle and bottom in *M. nudus*, and 26.9 and 2.4% in *P. lividus* (28 hpf and 24 hpf in Figures 5C1,C2). By 36 hpf, the proportion of larvae located in the middle and bottom increased in both species, reaching 54.2 and 38.7% in *M. nudus*, and 37.7 and 55.7% in *P. lividus*. At this stage, maximal differences between species were observed, with *M. nudus* larvae preferentially located in the middle part of the water column while most *P. lividus* larvae moved to the bottom (36 hpf in Figures 5C1,C2). By 40 hpf, most of the DA-treated larvae of both species (90.8% in *M. nudus* and 88.6% in *P. lividus*) were located at the bottom, with only a few (9.1 and 11.4% in *M. nudus* and *P. lividus*, respectively) in the middle. None of the 40 hpf DA-treated larvae remained at the top (40 hpf in Figures 5C1,C2). Thus, preincubation with dopamine resulted in a significant enhancement in larval downward swimming in both species, and the larvae’s ability to respond to DA increased with age. The experimental setup and summary of DA and 5-HT effects on different age larvae swimming patterns are presented in Figure 5G.

We then analyzed the number of DA-expressing and DA-positive neurons in representative larvae of different ages from the top, middle, and bottom groups. As described earlier (see section 2.1), DA-expressing neurons appeared at 36 hpf early pluteus, and their mean number increased with age in both species. An important finding was that at 36 hpf and 40 hpf, larvae in the bottom group contained the maximal number of DA-expressing neurons – 3-6 in 40 hpf in both species. In contrast, only 2–3 DA-expressing neurons were found in larvae from the middle group, and larvae from the top group either lacked or had just one such neuron (Figures 5D1,D2). After incubation in 5-HT, larvae were found only in the upper and middle parts of the water column and were absent at the bottom. However, again in this case, a greater number of DA-expressing neurons (up to 6) were found in larvae from the deeper layer (in this case, the middle layer), while only 1–4 neurons were present in the representatives of the top group (Figures 5E1,E2).

Treatment with DA allowed visualization of both DA-expressing and DA-uptaking neurons, collectively referred to as DA-positive neurons. Notably, at all ages, the maximal number of DA-positive neurons was found in representatives of the bottom group. The difference was most pronounced at the 36 hpf stage: 10–16 and 8–10 in representatives of *M. nudus* and *P. lividus* from the bottom group compared to 1–3 in representatives of the top group (Figures 5F1,F2). At 40 hpf, no larvae were located at the top. However, representatives from the middle group contained a smaller number of DA-positive neurons (8–10 in *M. nudus* and 6–8 in *P. lividus*) than those from the bottom (12–16 and 10–12, respectively; Figures 5F1,F2). In summary, both the number of DA-expressing neurons (in intact and 5-HT-treated groups) and DA-positive neurons (in DA-treated group) were maximal in individuals from the bottom group and minimal in individuals from the top group, maintaining this proportion in larvae of each examined stage.

In conclusion, we conducted a comparative analysis of the behavioral responses of *M. nudus* and *P. lividus* in the aforementioned experiments. In both species, there was an increase in the proportion of larvae predominantly situated at the bottom, coupled with a decrease in those positioned at the top as they matured (Figures 5H1,H2). Notably, *P. lividus* exhibited a more accelerated transition toward the bottom layer as their development progressed (Figure 5H1). The onset of DA-positive neurons occurred marginally later in *M. nudus*, while larvae of this species exhibited a swifter kinetics in the emergence of DA neurons (Figure 5I1). Conversely, apical 5-HT neurons manifested earlier and underwent more rapid development in *P. lividus*, with a consistently higher abundance observed at all developmental stages compared to *M. nudus* (Figure 5I2). The alteration in positioning in response to exogenous DA was highly similar across both species (Figure 5J1). However, the responsiveness to 5-HT was more pronounced at earlier developmental stages in *M. nudus*, converging to a similar level as *P. lividus* at the pluteus stage (Figure 5J2). When comparing between species, the response to DA did not demonstrate a direct correlation with the average number of DA-positive neurons, with developmental trajectories characteristic for each species (Figure 5K1). A similar pattern was evident for 5-HT neurons and their response to 5-HT (Figure 5K2). These findings underscore the species-specific characteristics inherent in the developmental dynamics of both neuron types investigated, as well as their association with swimming behavior throughout ontogeny, suggesting a nuanced interplay beyond mere neuron abundance.

## 3 Discussion

This study investigates the presence of post-oral neurons containing DA in sea urchin larvae, specifically *M. nudus* and *P. lividus*. Our findings emphasize the detectability of these neurons throughout development until the initiation of DA expression, highlighting their ability to uptake and store exogenous DA. The incorporation of the FaGlu express method for DA detection facilitated a crucial interspecies comparison. During normal sea urchin larval development, significant heterochrony was observed in both the quantity and timing of these neurons’ appearance in both studied sea urchin species. Furthermore, we identified a correlation between the vertical distribution of larvae in the water column and the number of DA and 5-HT neurons. Our exploration reveals similarities in both heterochrony and monoamine effects in both species, while also noting some species-specific features in terms of neuron quantity, vertical distribution, and the larva’s responsiveness to DA and 5-HT.

### 3.1 Comparison of dopaminergic elements in sea urchin larvae

The presence of dopaminergic neurons has been demonstrated at the four-arm pluteus stage in sea urchins *Psammechinus miliaris*, *Strongylocentrotus droebachiensis*, and *Echinocardium cordatum* (Ryberg, 1974; Bisgrove and Burke, 1987; Nezlin and Yushin, 1994). Authors utilized either histochemical techniques for catecholamine visualization or immunochemical detection of DA. Immunoreactivity against DA and histochemical fluorescence detection of catecholamine-containing cells revealed identical DA cell bodies and processes in *S. droebachiensis* (Bisgrove and Burke, 1987). Post-oral neurons also exhibited immunoreactivity to the essential enzyme for DA synthesis, tyrosine hydroxylase (TH), in *Lytechinus* var*iegatus* (Slota and McClay, 2018), and aromatic L-amino acid decarboxylase and TH in *Strongylocentrotus purpuratus* (Paganos et al., 2021). Regardless of the visualization methods used in all mentioned species, dopaminergic neurons were found concentrated in post-oral ganglia or groups of post-oral neurons, later appearing along the base of the arm and circumoral ciliary bands.

In the described sea urchin species, *M. nudus* and *P. lividus*, we also detected dopaminergic cells associated with arms. Using histochemical techniques for catecholamine visualization, we identified two groups of cells concentrated at the base of post-oral arms (latero-dorsal group, LDC) and oral lobe (latero-ventral group, LVC). Moreover, we demonstrated that these cells are capable of uptaking DA as early as at the gastrula stage. This suggests that these dopaminergic cells have already reached a differentiated state and express the DA transporter (an important part of the DA phenotype) earlier than they are able to synthesize DA. LDC and LVC groups of DA-uptaking neurons found in *M. nudus* and *P. lividus* correspond well with the post-oral neurons and lateral ganglion neurons described in other species of sea urchin larvae (Bisgrove and Burke, 1987; Slota and McClay, 2018; Paganos et al., 2021). Solitary cells situated in the anus and mouth area emerge subsequent to arm-associated dopaminergic cells in *M. nudus* and *P. lividus*, corresponding to the peripheral DA-containing cells in those regions (Bisgrove and Burke, 1987). Our results allow us to distinguish early differentiating post-oral groups of DA cells and late differentiating cells in the mouth and anus regions in the developing larvae of *M. nudus* and *P. lividus*.

Dopaminergic cells associated with post-oral arms were found in larvae of different sea urchin species (Chen and Adams, 2022). In *Eucidaris tribuloides*, *Arbacia punctulata*, *Lytechinus pictus*, and *Lytechinus* var*iegatus*, dopaminergic groups of cells present at pre-feeding gastrula and pluteus stages resemble LDC and LVC in *M. nudus* and *P. lividus*. At later feeding stages of these species, additional dopaminergic cells appear in the mouth and stomach region. Post-oral DA cells located at the base of post-oral arms, described in the pluteus of *L. variegatus* (Slota and McClay, 2018), and *S. purpuratus* (Paganos et al., 2021), align with the position of the cell bodies and cell projections along the arm edge, corresponding to the morphology of DA-containing and DA-uptaking cells in the corresponding areas of *M. nudus* and *P. lividus* pluteus. However, only late differentiating groups of DA-expressing cells in the mouth and stomach regions were present in *Echinarachnius parma*, *Dendraster excentricus*, *Encope michelini*, and *Echinometra lucunter* sea urchin larvae. The authors highlighted the interspecies variation in the number of DA cells, attributing this difference to the phenotypic plasticity of the post-oral arms (Chen and Adams, 2022). Nevertheless, they did not provide data concerning the number of DA cells at comparable developmental stages for each species, and they also did not address potential individual variations within the described species.

In our study, we demonstrated significant variations among individuals of the same species, which remained consistent for both *M. nudus* and *P. lividus* throughout all described stages from gastrula to 40 hpf pluteus. Notably, individuals lacking DA-containing cells coexisted with those containing 2 or even 6 DA cells. Additionally, the absence of cell appearance around the mouth prior to the differentiation of post-oral group cells was consistently observed. The observed differences in the distribution and timing of DA-positive neurons appearance within larvae of the same species prevailed over variations in dopaminergic neuron counts across different species (Chen and Adams, 2022). Our findings underscore the considerable variability in the morphology and timing of DA neurons among individuals within specific sea urchin species. The specific cell number within a particular group may not serve as a stable characteristic feature for the species. Importantly, in our experiments, all larvae were maintained under equal conditions and were not exposed to external stimuli. The identified variation in cell number suggests the presence of environmental-signal independent polymorphism in sea urchin larvae, arising from natural heterochrony in the development of the dopaminergic system. The expression of individual polymorphism in *M. nudus* and *P. lividus* equals or exceeds the interspecies differences described earlier (Chen and Adams, 2022). This underscores the need to consider such individual variations for the accurate interpretation of experimental work and evolutionary-developmental insights.

### 3.2 Post-oral dopaminergic neurons heterochrony and DA-mediated larval polymorphism in sea urchins

The preeminent form of larval polymorphism observed in sea urchins pertains to the varied post-oral arm lengths exhibited by pluteus larvae (Boidron-Metairon, 1988; Strathmann et al., 1992). This phenomenon ensues in response to nutritional cues and chemical determinants of predators (Miner, 2007; Barnes and Allen, 2023). The signaling transpires during pre-feeding larval stages and manifests in subsequent development. Previous investigations have demonstrated that DA signaling, mediated through a type-D2 receptor, governs this type of developmental plasticity (Adams et al., 2011). A correlation is observed, wherein heightened DA levels correspond to diminished arm length. Concurrently, it has been observed that the responsiveness to DA undergoes modifications with larval maturation (Kalachev and Tankovich, 2023). Noteworthy is the existence of analogous data for *M. nudus*, a species explored in this study (Kalachev, 2020).

Our study reveals that, even at pre-feeding stages and the absence of predators during the initial phases of larval development, an inherent polymorphism emerges in the quantity of DA-positive neurons, which serve as the primary source of DA signaling in ontogeny. Consequently, despite the lack of external environmental influences, the examined sea urchin larvae exhibit diversity in DA signaling levels.

Across each investigated developmental stage in both species, there is no statistically significant correlation between the quantity of dopaminergic neurons and the length of post-oral arms. This lack of correlation suggests that the observed polymorphism in neuron count does not manifest as a modulation of arm growth during the pre-feeding larval stages. It implies that the regulatory framework governing arm growth is likely more intricately structured than a direct influence of DA. Alternatively, the rate of DA release may be independently regulated, regardless of cellular quantity. This finding emphasizes the need for a more nuanced examination in future research to unravel the complexities of the regulatory mechanisms at play.

### 3.3 Heterochrony in the appearance of post-oral dopaminergic neurons and larval swimming patterns

**Figure 6:**
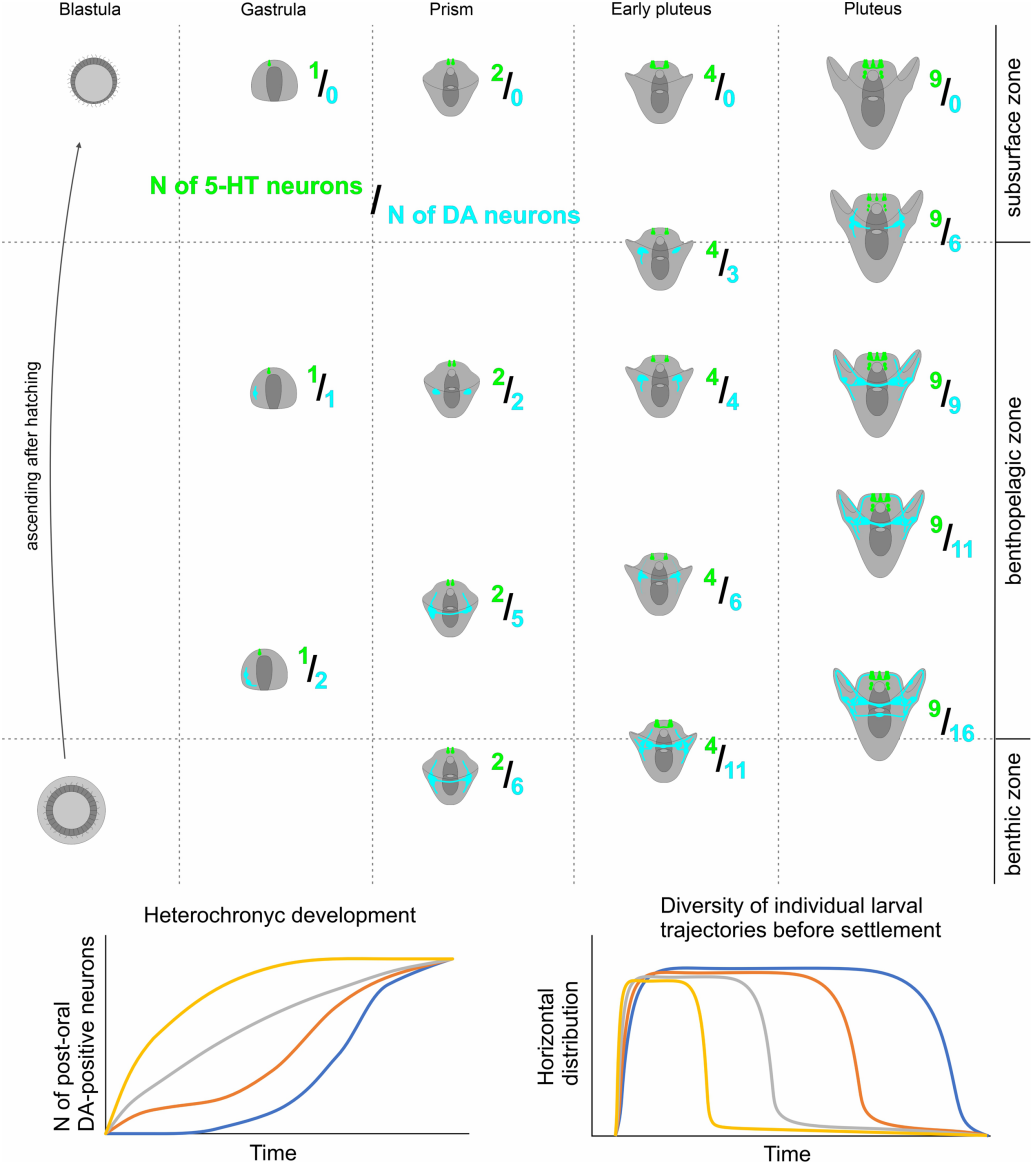
A proposed model for heterochronic development of larval DA-positive neurons and its impact on Individual swimming patterns in sibling echinoderm larvae. Overall, the quantity of both DA-positive neurons and 5-HT neurons increases as larval age progresses. However, the 5HT/DA-neuron ratio shows significant variability among larvae of the same age due to heterochronic polymorphism in the number of DA-positive neurons. Larvae with a higher count of DA-positive neurons tend to occupy deeper positions in the water column compared to their counterparts with fewer DA-positive neurons. This coordination of 5-HT and DA humoral regulation underlies a shift in larval behavior within a single generation, enabling polymorphic larval trajectories, and ensuring enhanced larval dispersal and population expansion.

Our findings indicate that heterochrony in the appearance of DA-positive neurons in sea urchin larvae of the same age significantly influences their vertical distribution within the water column. The development of more DA-expressing neurons enhances the larva’s ability to sink to the bottom. In contrast, 5-HT is associated with upward swimming, and the quantity of 5-HT-neurons determines the upper position of the larva. Consequently, in one generation, a limited number of larvae with preferential differentiation of the DA-positive neurons and respective downward ability appear at each developmental stage. We propose that the described synchrony of 5-HT-neurons differentiation, together with heterochrony in DA-positive neurons appearance, serves as a basis for the optimal vertical distribution of sibling animals. The possible model of vertical distribution of sea urchin species based on 5-HT/DA-positive neurons expression pattern polymorphism is presented in Figure 6.

Earlier, interspecies heterochrony in the appearance of 5-HT neurons has been described between the larvae of planktotrophic and lecithotrophic sea urchin species (Bisgrove and Raff, 1989). The alteration in the time of nervous system development between direct and indirect developers implicates heterochrony in cellular differentiation as an important component of adaptation. Generally, heterochrony, as an evolutionary shift in the relative time of certain structure formations, is widely distributed among developmental programs and serves as one of the sources for evolutionary changes (Kaufman and Raff, 1983; Gould, 1985; Smith, 2003), especially in echinoderms (Parks et al., 1988; Bisgrove and Raff, 1989). Our finding of adaptational heterochrony in nervous system development within the sibling larvae exemplifies neuronal plasticity, potentially underlying evolutionary adaptations within a particular species. The possible cellular mechanism of this phenomenon requires further investigation.

We observed a correlation between the number of DA-positive and 5-HT-neurons in larvae and their swimming pattern, driven by ciliary beating. Both DA and 5-HT are well-established regulators of cilia activity, and their roles in the control of sea urchin larval swimming have been extensively studied. Previous research has demonstrated opposing effects of 5-HT and DA on the ciliary beating rate of arm bands’ cilia, with 5-HT reducing the beat period and DA prolonging it (Wada et al., 1997). Our experimental results, revealing a positive correlation between the differentiation of DA-positive elements and larval downward swimming ability, are in line with existing data. A higher number of DA-positive neurons potentially can synthesize and release more DA, leading to a reduction in ciliary beating rate. Consequently, larvae lose their swimming ability and sink. 5-HT counteracts this downward swimming by increasing swimming ability through elevated ciliary beating (5-HT reduces the beat period of cilia). Therefore, the balance between the DA and 5-HT systems during development can determine the prevailing position of the larva in the water column.

The majority of time during which larval dispersion occurs in sea urchins is spent during the feeding larval stage. However, despite the simplicity of the nervous system organization and the low number of neurons in the early larval development stages, pre-feeding sea urchin larvae exhibit quite complex changes in swimming patterns in response to changing environmental conditions. Starting from the late blastula stage, sea urchin larvae demonstrate responses such as changing their position in the water column in response to alterations in light direction and intensity (Yaroslavtseva et al., 2002; Yaguchi et al., 2022). By the prism stage, larvae have the ability to move between layers of water with different salinity, choosing the optimal layer (Yaroslavtseva et al., 2002). Even early larvae shortly after hatching demonstrate stabilized rotation direction and stable orientation of the rotational axis relative to the direction of movement (Maruyama, 1981). These and many other adaptive features of early behavior require the development of specific neuronal systems. Thus, the polymorphism we observed may be part of the regulation system drawing the larval complex behavior even at early in pre-feeding stages.

The early stages of neural development play a crucial role in determining the developmental trajectory and, consequently, the character of complex adaptation throughout an animal’s life. DA serves as one of the essential regulators of nervous system development. In vertebrates, altered DA signaling can influence the proliferation, migration, and differentiation of specific subpopulations of neurons, thereby impacting the further development of the nervous system (Money and Stanwood, 2013). Catastrophic development is a characteristic feature of sea urchins, and the development of the adult nervous system in echinoderms, in general, remains poorly understood. However, the polymorphism we observed in the development of DA-positive post-oral neurons may also be relevant at later stages of nervous system formation in these animals.

The ability of planktonic larvae to disperse is a crucial factor for the ecological niche of a species (Alexandridis et al., 2017). Planktonic larvae swimming in the upper layers of water can be carried by sea currents for long distances, and the longer the larva remains an active swimmer, the further it can be carried away from the residence of the benthic parental specimens (Jablonski and Lutz, 1983). In temperate sea urchin species, this planktonic stage generally lasts from 2 to 6 weeks (Thorson, 1961), and during this period, an ocean current of only 0.5 km/h could carry a larva for 150–550 km (Scheltema, 1977). Larvae of some tropical species can remain in the plankton up to 6 months or more, traversing trans-oceanic distances (Edmunds, 1977; Scheltema, 1977). On the other hand, some polar and deep-sea species demonstrate demersal larvae that swim or crawl in the near-bottom water layer until settlement (Pearse, 1969). In high latitudes, this dispersal mode appears to be an adaptive compromise, retaining some of the advantages of free-swimming larvae while reducing mortality by keeping larvae out of dangerous surface waters (Pearse, 1969). Benthic dispersal at any latitude would expose larvae to a more stable benthic food resource and a temperature-salinity regime compared to the seasonally and diurnally more variable conditions at the sea surface, particularly in nearshore waters (Levinton, 1974; Hendler, 1977; McCall, 1978). Polymorphism in the appearance of DA-positive neurons and mediated diversity of swimming patterns may be a mechanism for the emergence of internal polymorphism, expanding the ecological plasticity of larvae within the species.

### 3.4 Post-oral dopaminergic neurons in sea urchin, and the evolution of endocrine system

Recent studies have revealed that post-oral dopaminergic neurons express the enzyme for acetylcholine synthesis and a cocktail of neuropeptides (Slota and McClay, 2018; Paganos et al., 2021). This demonstrates that post-oral neurons can influence ciliary beating in a more intricate manner than merely through the frequency of beats, determining finer patterns of larval behavior. It happens via neurotransmitter receptors that are directly located on the cilia (Katow et al., 2010). Moreover, processes from serotoninergic and dopaminergic neurons do not directly extend to each ciliary cell, forming synapses. Thus, 5-HT and DA act on cilia rather as humoral endocrine regulators.

Hypotheses about the origin of pancreatic β-cells from neurons have been proposed earlier based on the proximity of their neurogenic marker expression profile (Eberhard, 2013). Recent studies have shown that DA-positive post-oral neurons in sea urchins express Pdx1 (one of the master-regulators for β-cells differentiation) and a set of markers that indicate their homology with β-cells in the mammalian pancreas (Perillo et al., 2018; Paganos et al., 2021). These findings have sparked discussions about the evolution of the endocrine system and its conservation across different animal phyla.

Our findings suggest the presence of a regulatory mechanism governing the number of post-oral neurons during the development of sea urchins. Typically, the regulation through cell number and mass is more characteristic of endocrine organs, while adaptive physiological regulations through intricate cell interactions are more typical for the nervous system (Leroith et al., 1986). Pancreatic β-cells adhere to this pattern, featuring a complex system for controlling cell mass (Bouwens and Rooman, 2005). Consequently, this endocrine organ is distinguished by the determination of developmental trajectories, influencing various aspects of feeding behavior and adaptive physiological traits in adult life (Kim et al., 2010; Castell et al., 2022). Our discovery reveals that in sea urchins, the post-oral neurons is a part of a complex neuroendocrine regulator, resembling its operation in vertebrates. Notably, the natural polymorphism in the number of post-oral neurons determine feeding behavior-related aspects of larval development. This implies that this system was present at the level of the common ancestor of ambulacrarians and chordates in its fully functional form, rather than as distinct components. Moreover, in addition to cellular type homology, there is a direct functional continuity observed in this system between sea urchins and vertebrates.

Our study primarily focuses on serotoninergic and dopaminergic cells as humoral regulators. In vertebrates, 5-HT serves a partially direct endocrine function, while DA has largely relinquished this role over the course of evolution. However, the regulation of cell mass in pancreatic β-cells is still intertwined with these ancient monoaminergic regulatory systems. β-cells express 5-HT receptors, and 5-HT increases their proliferation in pubertal islets (Castell et al., 2022). DA synthesis is absent in β-cells in vertebrates. Nevertheless, DA is synthesized by α cells, while β-cells express the DA D2 receptor, acting as a positive regulator of the cell mass of β-cells (Sakano et al., 2016). Thus, despite their specialization in neurotransmitter function, monoamines within this endocrine system retain their ancient, overarching integrative role, governing the intricate interplay of feeding behavior and physiological processes at the population adaptation level.

## 4 Conclusion and future directions

In conclusion, our research not only advances the understanding of sea urchin larval development but also contributes to broader discussions on the evolutionary and ecological implications of neuroendocrine adaptations. The intricate coordination between the 5-HT and DA systems, along with the observed shifts in larval behavior within a single generation, underscores the adaptability of these organisms to optimize ecological success. Future investigations should delve deeper into the cellular and molecular mechanisms orchestrating the phenomenon of spontaneous intersibling polymorphism in DA cells, providing a more nuanced understanding of the evolutionary strategies at play.

## 5 Materials and methods

### 5.1 Animal handling and larval culture

Adult *Mesocentrotus nudus* (Agassiz, 1864) were collected from Peter the Great Bay in the Sea of Japan at the “Vostok” Biological Station, Zhirmunsky Institute of Marine Biology of the Far East Branch of the Russian Academy of Sciences. Adult *Paracentrotus lividus* (Lamarck, 1816) were obtained from the Mediterranean Sea near Paphos, Cyprus, and from the marine station of Endoume, France (Station Marine d’Endoume IMBE, Marseille, France). Spawning was induced by intracoelomic injection of 0.5 M KCl. Eggs were collected and fertilized in filtered seawater (FSW) at a concentration of 1800–2,500 eggs/mL to ensure a uniform monolayer at the bottom of a glass vessel with a diameter of 30 cm and a height of 15 cm, filled with 2 L of FSW. Two to three drops of concentrated sperm were added to the water and gently mixed. After 20 min for fertilization, the FSW with sperm was replaced with 5 L of clean FSW. The quality of fertilization was assessed for each experiment and ranged from 96 to 100%. After 18 h, the hatched blastulae were collected from the water surface and gently transferred to a 5 L glass beaker filled with FSW. Directed airflow to the water surface (without gurgling) was used to create a circular current of water in the beaker. Embryos and larvae were cultured at 18°C until they reached the gastrula, prism, two-armed early pluteus, and pluteus stages, and were then used for morphological studies and swimming experiments. The larvae did not receive food during the culturing period. For experiments, larvae were concentrated from the 5 L beaker using a 100 μm net.

Both *M. nudus* and *P. lividus* have planktotrophic larvae, exhibiting high similarity in larval general morphology throughout their development. Gastrulation is completed at approximately 28 hpf. By the prism stage (32 hpf), larvae have developed a digestive system comprising the mouth, esophagus, stomach, and anus. The first pair of arms (post-oral arms) begins to form during the prism stage and persists into the early pluteus (36 hpf) and pluteus (40 hpf) stages. Ciliary bands, crucial for larval swimming, develop along the edges of the arms and the oral lobe. Simultaneously, the apical organ differentiates at the apical pole of the oral lobe during the described stages (Bisgrove and Raff, 1989; Paganos et al., 2021). Spawned adults and larvae not used for experiments were returned to their natural environment.

### 5.2 Histochemical detection of DA-positive cells

**Figure 7:**
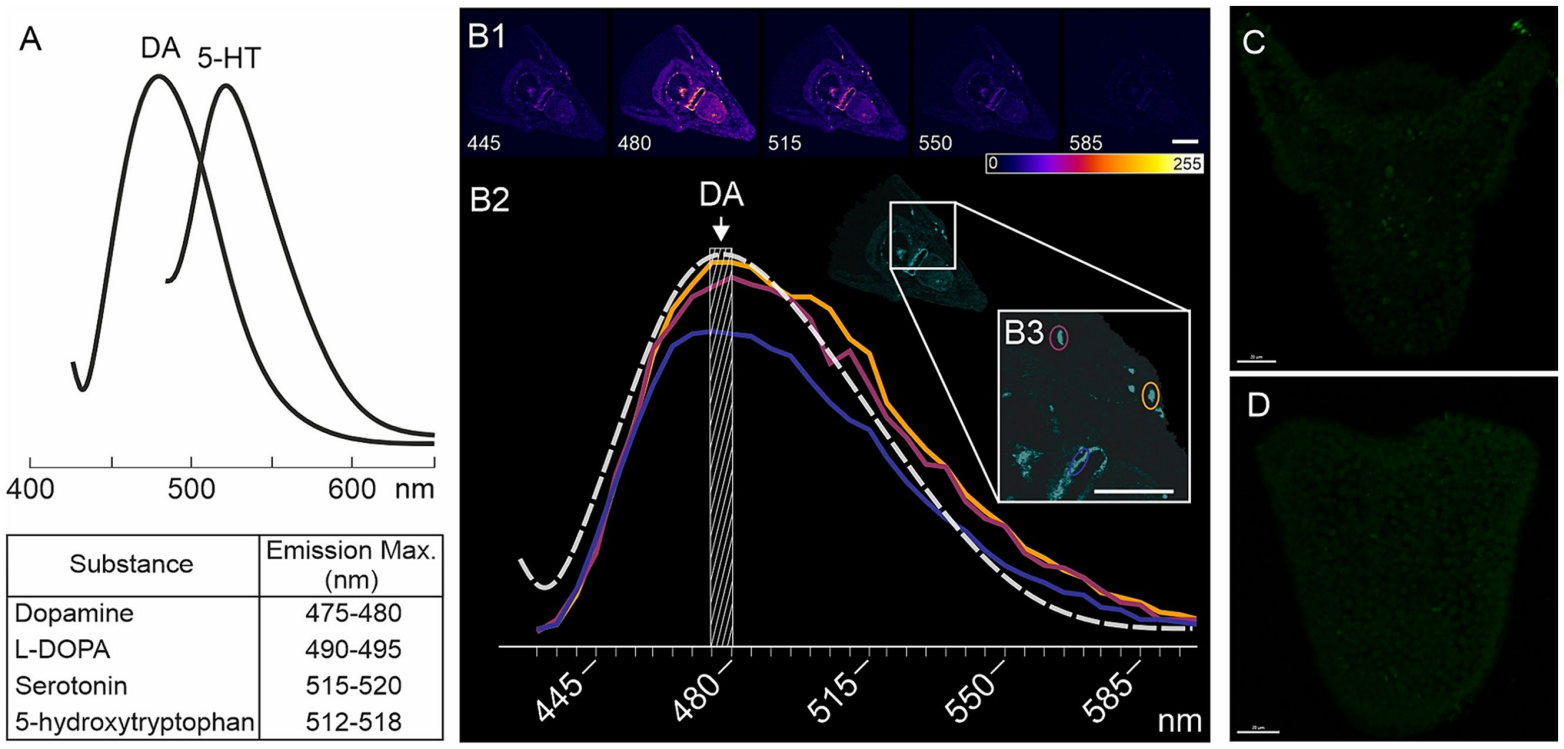
Characteristics of FaGlu-induced fluorescence and negative controls for 5-HT immunoreactivity. **(A)** – Sample of emission spectrum for biogenic amines following FaGlu histochemical reaction (adapted from Furness et al., 1977). **(B1)** – FaGlu reaction samples in pluteus larvae at various emission wavelengths with emission at 405 nm. **(B2)** – Emission spectrum of selected neurons correlates with the emission spectrum of dopamine. **(C,D)** – Immunohistochemical reaction with omission of primary antibody against 5-HT in prism and pluteus larvae. Color-coding from 0 (dark blue) to 255 (white) indicate brightness intensity in selected excitation wavelength. Scale bars: **(B1,B3,C,D)** — 20 μm.

Larvae from four different fertilizations of *M. nudus* and three different fertilizations of *P. lividus* were used for histochemical detections of catecholamines. Larvae of respective ages (24, 28, 32, 36 and 40 hpf) were concentrated from a 5 L beaker using a 100 μm net and transferred to 2 mL Eppendorf tubes using Paster pipette. Collected larvae of each age were prefixed with the addition of a few drops of 4% paraformaldehyde (PFA). The sea water was removed and larvae underwent fixation in mixture of glutaraldehyde and paraformaldehyde. For the histochemical detection of catecholamines, particularly dopamine (DA), we utilized a mixture of 0.5% glutaraldehyde and 4% paraformaldehyde in filtered seawater (FSW) with 30% sucrose (FaGlu). Samples were stored in the FaGlu mixture at 4°C for a period ranging from 1 day to 1 month until examination. Prior to imaging, samples were mounted on glass slides, excess FaGlu was carefully removed, and the glass slides were stored in a dry, dark place at room temperature for 12 h to dry. After drying, the samples were coated with paraffin oil and covered with a coverslip. Upon completion of the reaction the larvae were examined under a fluorescent microscope Leica DMI-6000 with appropriate filter set allowing to recognize specific blue-green fluorescence of catecholamines. Larvae were systematically viewed across the entire field of view and the cells with specific fluorescence were counted for 20–30 individuals of each age for *M. nudus* and *P. lividus* from each fertilization. Larvae with orientation allowing to visualize maximal number of neurons were selected to scanned for further analysis of DA-positive neurons position and illustrations. Selected samples were examined using a Leica TCS SP5 confocal laser scanning microscope with excitation at 405 nm and emission filters set at 475–485 nm (Zoological Institute RAS and Saint-Petersburg State University, Saint Petersburg, Russia). This combination of excitation and emission filters enables the specific detection of fluorescent products derived from dopamine (Furness et al., 1977). The spectra of biogenic amines are presented in Figure 7A, and the emission characteristics for DA-positive neurons are depicted in Figures 7B1–B3. This spectrum corresponds to the blue-green fluorescence of neurons visible under Leica DMI-6000.

### 5.3 Immunohistochemical detection of 5-HT-containing cells

The portion of larvae that were prefixed after concentration for detection of catecholamines (see 3.2) was selected for immunochemical detection of serotonin (5-HT). Thus, the larvae from the same fertilization bath were proceeds for both histochemical detection of catecholamines and immunochemical detection of 5-HT. To detect 5-HT-containing neurons, larvae were fixed in 4% paraformaldehyde in FSW at the corresponding developmental stages. Prior to antibody treatment, embryos were washed three times with 0.01 M phosphate-buffered saline (PBS) pH 7.4 containing 0.5% Triton X-100 (PBS-TX), and then incubated for 12–24 h at 4°C with rabbit antibodies against 5-HT (rabbit, Immunostar, Cat #20080) diluted 1:1000 in 0.5% PBS-TX. Following incubation with the primary antibodies, the samples were washed three times with 0.1% PBS-TX and treated with Alexa Fluor 488-tagged goat anti-rabbit IgG antibodies (Thermo Fisher, Cat # A-11008), diluted 1:800 in 0.1% PBS-TX, for 6–12 h at 4°C. Subsequently, the samples were washed three times with PBS and immersed in 70% glycerol. Examination of the samples was performed using a confocal laser scanning microscope Leica TCS SP5 (Leica, Germany) with appropriate wavelength filter configuration settings (IDB RAS, Moscow, Russia). 15–20 individuals were viewed under Leica DMI-6000 microscope and 5-HT cells were counted. Larvae with orientation allowing to recognize the maximal number of neurons were selected for scanning for illustrations. Image analysis was conducted using Fiji software (Schindelin et al., 2012). Negative controls, including omission of the primary antibody, demonstrated no specific staining (Figures 7C,D).

### 5.4 Swimming and neuron counting assays

**Figure 8:**
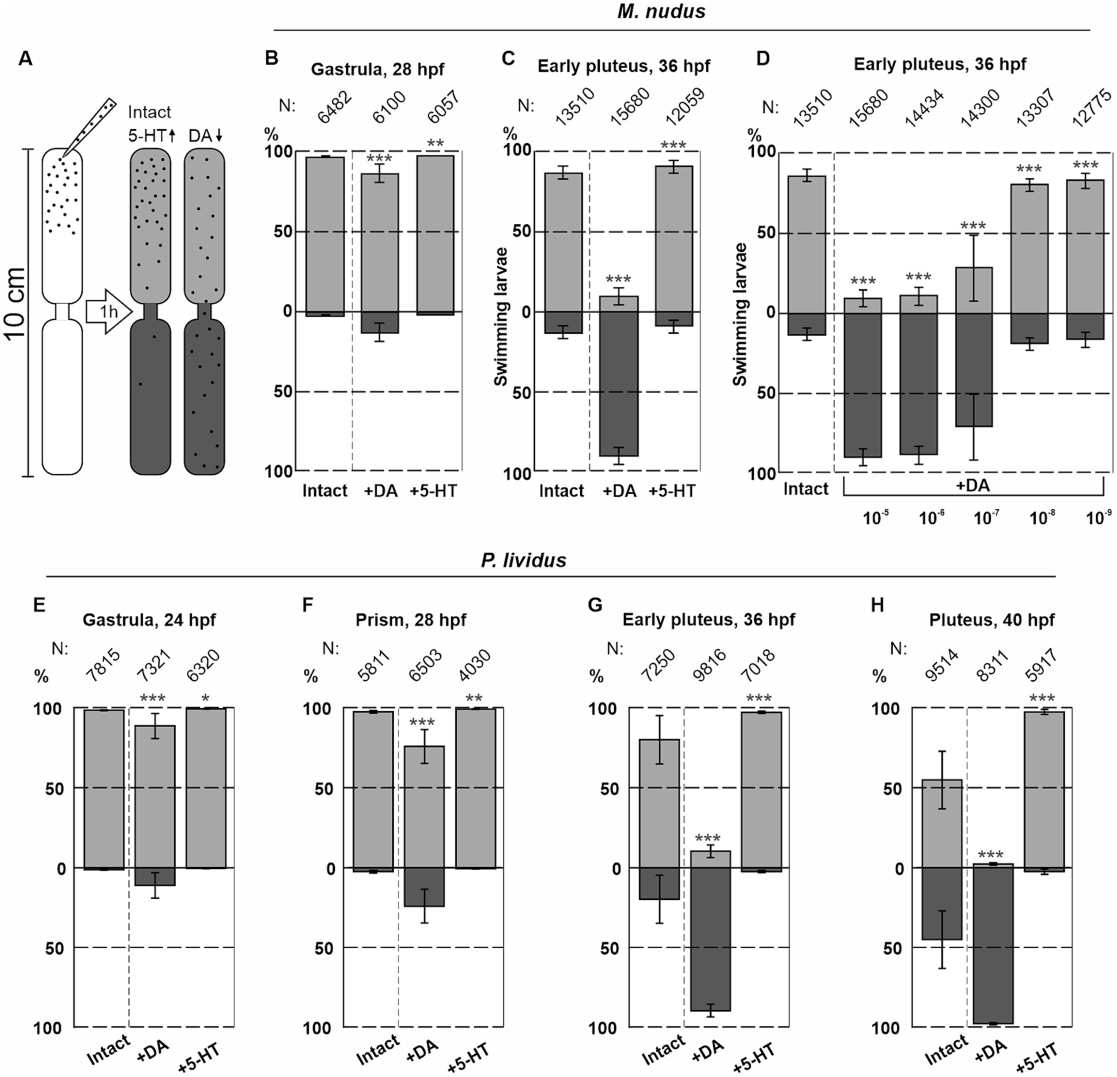
Syringe behavioral assays to assess the impact of DA and 5-HT on larval swimming at different ages. **(A)** – Schematic representation of the experimental setup. **(B,C)** – The numbers of upward and downward swimming gastrula and early pluteus larvae of *M. nudus* in response to DA and 5-HT. **(D)** – Downward swimming of *M. nudus* early pluteus in response to DA is dose-dependent, with 1  selected as optimal concentration required to affect swimming. **(E–H)** – Application of 5-HT results in upward swimming in *P. lividus* larvae of all ages, while DA affects downward swimming ability stage-dependently. Notably, the impact of DA increases with larval age from gastrula to pluteus. N represents the number of larvae. Five independent replications for each test were performed. Chi-square test results comparing to the respective intact control points are provided. Statistical significance: *p* < 0.05: *, *p* < 0.01: **, *p* < 0.001: ***.

Seven fertilizations were conducted using two females and one male for *M. nudus*, and six fertilizations using the same protocol were performed for *P. lividus*. The larvae were incubated in plastic Petri dishes with a diameter of 4 cm, and 5-HT or DA at concentrations ranging from 0.1 μM to 0.01 mM were added to the Petri dishes. The larvae were allowed to incubate for 30 min, with filtered seawater (FSW) used for the control group. Preliminary experiments utilized a setup previously described by Yaguchi and Katow (2003) (see Figure 8A). This setup comprised two plastic tubes (1 cm in diameter and 5 cm in length) placed one above the other with a narrow channel (1 mm in diameter) between them. The lower tube and the channel between the tubes were filled with FSW, while 2 mL of the incubation solution containing larvae from Petri dishes were transferred to the upper tubes. The setup was then placed in a dark environment for 1 h. After the swimming assay, the upper and lower parts of the setup were separated, and water with larvae was transferred to 9 cm Petri dishes. The number of larvae in the upper and lower portions was counted under a dissecting microscope. All experimental procedures with larvae were conducted at 18°C.

The number of downward swimming larvae increased with larval age from the gastrula to the pluteus stage for both species (see Figures 8B,C,E–H). *M. nudus* early pluteus demonstrated a dose-dependent downward swimming reaction in response to DA application (Figure 8D). Based on these preliminary experiments, DA and 5-HT at concentrations of 1 μM were selected as an optimal for the further experiments.

For the swimming experiments, we developed and constructed an experimental setup comprising several vertical columns measuring 5 cm in diameter and 1 m in height. Each column consisted of a soft polyethylene tube with a closed bottom and an open upper section. Prior to the experiments, the tubes were filled with filtered seawater (FSW) and positioned vertically in a dimly lit environment. Experimental larvae from their respective treatment groups (DA-treated and 5-HT-treated) were rinsed in FSW and gently introduced into the upper portion of the column. Following a 1-hour swimming assay, the flexible plastic tubes were clamped at two locations: 25 cm from the top and 25 cm from the bottom, effectively dividing the water column into three segments: top, middle, and bottom (for further details, refer to Figure 5G). Subsequently, water containing larvae was collected from each segment and transferred to 0.5 L glass containers. The larvae were fixed by adding a few drops of 4% paraformaldehyde (PFA). Once immobilized, the larvae were transferred from the glass containers to 9 cm Petri dishes and enumerated under a dissecting microscope. By simultaneously and swiftly collecting larvae from the three segments of the column, we achieved the most precise determination of the proportion of larvae exhibiting each type of swimming behavior at each developmental stage: top, middle, and bottom. All swimming assays were performed in the absence of light to mitigate the potential influence of illumination on larval behavior (Yaguchi et al., 2022). The described experimental setup ensured that the conditions closely resembled natural environmental conditions: the relatively wide diameter and height of the columns minimized the effects of capillary force, and larvae could only access any part of the column through their own movement.

To compare the larval swimming ability and the number of DA-positive neurons, we performed experiments using two different fertilizations for *M. nudus* and *P. lividus*, with two females and one male used in each replicate. After performing swimming experiments, we collected water containing larvae from the top, middle, and bottom portions of the water column as described above. To ensure unbiased selection, larvae were randomly sampled from various regions of the bottom of the Petri dish. Subsequently, all selected larvae underwent fixation for the FaGlu reaction. Upon completion of the reaction, the larvae were carefully positioned on a slide and systematically scanned across the entire field of view. The larvae with orientations allowing visualization of the maximal number of specific neurons were selected for scanning. Neurons in 10 individuals from each swimming group were counted for each age in intact, DA-treated, and 5-HT-treated experimental groups for both *M. nudus* and *P. lividus*.

### 5.5 Length measurements

Measurements of post-oral arm length (AL) and body length (BL) in larvae of different ages and corresponding DA-expressing and DA-positive neurons were performed in an additional fertilization of *M. nudus* and *P. lividus*. Twenty to thirty individuals of examined ages (32, 36, 40 hpf for *M. nudus* and 28, 32, 36, and 40 hpf for *P. lividus*) from intact and DA-treated groups were processed for FaGlu staining. Only individuals with an unambiguous ventral orientation and clearly visible DA-positive neurons were used for counting. All measurements were based on projections of confocal images for each larva. The schematic representation of AL and BL measurements can be observed in Figure 4D. Each individual larva counted is presented as a separate point in the graph for intact and DA-treated experimental groups (Figures 4G–L).

### 5.6 Statistical analysis

Experiments were conducted with three pairs of non-related male–female crosses. The distribution of swimming larvae in response to 5-HT and DA, compared with the control, was analyzed using the Chi-square test. Chi-square test calculations were performed using an interactive tool for chi-square tests of goodness of fit and independence.^1^ Replicates were combined for chi-square estimations. The Spearman correlation test was conducted using XLStat, and Bonferroni correction of *p*-values for multiple (three replicates) comparisons was applied. *p*-values less than 0.05 were considered statistically significant. Variances were calculated in centered samples using Microsoft Excel 2016. Graphs were generated with GraphPad Prism v. 8 and Microsoft Excel 2016, and mean ± SEM values are provided.

## Data availability statement

The original contributions presented in the study are included in the article/supplementary material, further inquiries can be directed to the corresponding authors.

## Author contributions

AO: Conceptualization, Investigation, Methodology, Visualization, Writing – original draft, Writing – review & editing. MK: Investigation, Writing – review & editing. MS: Investigation, Resources, Writing – review & editing. VS: Funding acquisition, Resources, Writing – review & editing. EV: Conceptualization, Funding acquisition, Investigation, Methodology, Project administration, Resources, Supervision, Writing – original draft, Writing – review & editing. EI: Conceptualization, Data curation, Formal analysis, Investigation, Methodology, Project administration, Supervision, Visualization, Writing – original draft, Writing – review & editing.

## Footnotes

^1^ https://www.quantpsy.org/chisq/chisq.htm

## References

[ref1] Adams D. K. Sewell M. A. Angerer R. C. Angerer L. M. (2011). Rapid adaptation to food availability by a dopamine-mediated morphogenetic response. Nat. Commun. 2:592. doi: 10.1038/ncomms1603, PMID: 22186888 PMC3992878

[ref2] Agatsuma Y. (2020). “Chapter 34 - *Mesocentrotus nudus*” in Developments in aquaculture and fisheries Science Sea urchins: Biology and ecology. ed. Lawrence J. M. (Amsterdam, Netherlands: Elsevier), 627–641.

[ref3] Alexandridis N. Bacher C. Desroy N. Jean F. (2017). Building functional groups of marine benthic macroinvertebrates on the basis of general community assembly mechanisms. J. Sea Res. 121, 59–70. doi: 10.1016/j.seares.2017.01.007

[ref4] Barnes D. K. Allen J. D. (2023). Predators induce phenotypic plasticity in echinoderms across life history stages. Biol. Bull. 244, 103–114. doi: 10.1086/725633, PMID: 37725697

[ref5] Bisgrove B. W. Burke R. D. (1987). Development of the nervous system of the pluteus larva of *Strongylocentrotus droebachiensis*. Cell Tissue Res. 248, 335–343. doi: 10.1007/BF00218200

[ref6] Bisgrove B. W. Raff R. A. (1989). Evolutionary conservation of the larval serotonergic nervous system in a direct developing sea urchin. Develop. Growth Differ. 31, 363–370. doi: 10.1111/j.1440-169X.1989.00363.x 37281459

[ref7] Boidron-Metairon I. F. (1988). Morphological plasticity in laboratory-reared echinoplutei of *Dendraster excentricus* (Eschscholtz) and *Lytechinus variegatus* (Lamarck) in response to food conditions. J. Exp. Mar. Biol. Ecol. 119, 31–41. doi: 10.1016/0022-0981(88)90150-5

[ref8] Boudouresque C. F. Verlaque M. (2020). “Chapter 26- *Paracentrotus lividus*” in Developments in aquaculture and fisheries Science Sea urchins: Biology and ecology. ed. Lawrence J. M. (Amsterdam, Netherlands: Elsevier), 447–485.

[ref9] Bouwens L. Rooman I. (2005). Regulation of pancreatic beta-cell mass. Physiol. Rev. 85, 1255–1270. doi: 10.1152/physrev.00025.2004 16183912

[ref10] Burke R. D. (1983). Development of the larval nervous system of the sand dollar, *Dendraster excentricus*. Cell Tissue Res. 229, 145–154. doi: 10.1007/BF00217887, PMID: 6831540

[ref11] Castell A.-L. Goubault C. Ethier M. Fergusson G. Tremblay C. Baltz M.. (2022). β cell mass expansion during puberty involves serotonin signaling and determines glucose homeostasis in adulthood. JCI Insight 7:854. doi: 10.1172/jci.insight.160854, PMID: 36107617 PMC9675460

[ref12] Chen E. Adams D. (2022). The evolution of neurosensation provides opportunities and constraints for phenotypic plasticity. Sci. Rep. 12:11883. doi: 10.1038/s41598-022-15583-y, PMID: 35831328 PMC9279360

[ref13] Conzelmann M. Offenburger S.-L. Asadulina A. Keller T. Münch T. A. Jékely G. (2011). Neuropeptides regulate swimming depth of *Platynereis* larvae. Proc. Natl. Acad. Sci. 108, E1174–E1183. doi: 10.1073/pnas.1109085108, PMID: 22006315 PMC3219130

[ref14] D’Aniello E. Paganos P. Anishchenko E. D’Aniello S. Arnone M. I. (2020). Comparative neurobiology of biogenic amines in animal models in deuterostomes. Front. Ecol. Evol. 8:322. doi: 10.3389/fevo.2020.587036

[ref15] Degnan S. M. Degnan B. M. (2006). The origin of the pelagobenthic metazoan life cycle: what’s sex got to do with it? Integr. Comp. Biol. 46, 683–690. doi: 10.1093/icb/icl028, PMID: 21672778

[ref16] DeWitt T. J. Sih A. Wilson D. S. (1998). Costs and limits of phenotypic plasticity. Trends Ecol. Evol. 13, 77–81. doi: 10.1016/S0169-5347(97)01274-3 21238209

[ref17] Eberhard D. (2013). Neuron and beta-cell evolution: learning about neurons is learning about beta-cells. BioEssays 35:584. doi: 10.1002/bies.201300035, PMID: 23575922

[ref18] Edmunds M. (1977). Larval development, oceanic currents, and origins of the opisthobranch fauna of Ghana. J. Molluscan Stud. 43, 301–308. doi: 10.1093/oxfordjournals.mollus.a065385

[ref19] Furness J. B. Costa M. Wilson A. J. (1977). Water-stable fluorophores, produced by reaction with aldehyde solutions, for the histochemical localization of catechol- and indolethylamines. Histochemistry 52, 159–170. doi: 10.1007/BF00492292, PMID: 406252

[ref20] Goldberg J. I. Rich D. R. Muruganathan S. P. Liu M. B. Pon J. R. Tam R.. (2011). Identification and evolutionary implications of neurotransmitter-ciliary interactions underlying the behavioral response to hypoxia in *Lymnaea stagnalis* embryos. J. Exp. Biol. 214, 2660–2670. doi: 10.1242/jeb.053009 21795561

[ref21] Gould S. J. (1985). Ontogeny and phylogeny. Cambridge, MA: Belknap Press of Harvard University Press.

[ref22] Hendler G. (1977). Development of *Amphioplus abditus* (Verrill) (Echinodermata: Ophiuroidea): I. Larval biology. Biol. Bull. 152, 51–63. doi: 10.2307/1540726, PMID: 556962

[ref23] Jablonski D. Lutz R. A. (1983). Larval ecology of marine benthic invertebrates: paleobiological implications. Biol. Rev. 58, 21–89. doi: 10.1111/j.1469-185X.1983.tb00380.x

[ref24] Kalachev A. (2020). Effect of dopamine on early larvae of sea urchins, *Mesocentrotus nudus* and *Strongylocentrotus intermedius*. Exp. Zool. B Mol. Dev. Evol. 334, 373–380. doi: 10.1002/jez.b.23001, PMID: 32902119

[ref25] Kalachev A. Tankovich A. (2023). The dopamine effect on sea urchin larvae depends on their age. Dev. Grow Dif. 65, 120–131. doi: 10.1111/dgd.12839, PMID: 36645274

[ref26] Katow H. Suyemitsu T. Ooka S. Yaguchi J. Jin-Nai T. Kuwahara I.. (2010). Development of a dopaminergic system in sea urchin embryos and larvae. J. Exp. Biol. 213, 2808–2819. doi: 10.1242/jeb.042150, PMID: 20675551

[ref27] Kaufman T. C. Raff R. A. (1983). Embryos, genes, and evolution: The developmental-genetic basis of evolutionary change. New York: Macmillan.

[ref28] Kim H. Toyofuku Y. Lynn F. C. Chak E. Uchida T. Mizukami H.. (2010). Serotonin regulates pancreatic β-cell mass during pregnancy. Nat. Med. 16, 804–808. doi: 10.1038/nm.2173, PMID: 20581837 PMC2921604

[ref29] Kuang S. Doran S. A. Wilson R. J. A. Goss G. G. Goldberg J. I. (2002). Serotonergic sensory-motor neurons mediate a behavioral response to hypoxia in pond snail embryos. J. Neurobiol. 52, 73–83. doi: 10.1002/neu.10071, PMID: 12115895

[ref30] Kuang S. Goldberg J. I. (2001). Laser ablation reveals regulation of ciliary activity by serotonergic neurons in molluscan embryos. J. Neurobiol. 47, 1–15. doi: 10.1002/neu.1011, PMID: 11257609

[ref31] Leroith D. Delahunty G. Lynn Wilson G. Roberts C. T. Shemer J. Hart C.. (1986). “Evolutionary aspects of the endocrine and nervous systems” in Proceedings of the 1985 Laurentian hormone conference recent Progress in hormone research. ed. Greep R. O. (Boston: Academic Press), 549–587. 10.1016/b978-0-12-571142-5.50017-3 3090659

[ref32] Levinton J. S. (1974). Trophic group and evolution in bivalve molluscs. Palaeontology 17, 579–585.

[ref33] Maruyama Y. K. (1981). Development of swimming behavior in sea urchin embryos. J. Exp. Zool. 215, 163–171. doi: 10.1002/jez.1402150205

[ref34] McCall P. L. (1978). “Spatial-temporal distributions of long island sound infauna: the role of bottom disturbance in a nearshore marine habitat” in Estuarine interactions. ed. Wiley M. L. (Cambridge, MA: Academic Press), 191–219.

[ref35] Miner B. G. (2007). Larval feeding structure plasticity during pre-feeding stages of echinoids: not all species respond to the same cues. J. Exp. Mar. Biol. Ecol. 343, 158–165. doi: 10.1016/j.jembe.2006.11.001

[ref36] Mogami Y. Watanabe K. Chieko O. Akemi K. Baba S. A. (1992). Regulation of ciliary movement in sea urchin embryos: dopamine and 5-HT change the swimming behaviour. Comp. Biochem. Physiol. C Pharmacol. Toxicol. 101, 251–254. doi: 10.1016/0742-8413(92)90269-D

[ref37] Money K. M. Stanwood G. D. (2013). Developmental origins of brain disorders: roles for dopamine. Front. Cell. Neurosci. 7:260. doi: 10.3389/fncel.2013.00260, PMID: 24391541 PMC3867667

[ref38] Nezlin L. P. Yushin V. V. (1994). The digestive tract of the echinopluteus of *Echinocardium cordatum* (Echinodermata, Echinoida): its ultrastructure and innervation. Can. J. Zool. 72, 2090–2099. doi: 10.1139/z94-280

[ref39] Nielsen C. (2009). How did indirect development with planktotrophic larvae evolve? Biol. Bull. 216, 203–215. doi: 10.1086/BBLv216n3p203, PMID: 19556589

[ref40] Nielsen C. (2012). Animal evolution: Interrelationships of the living phyla. Oxford: Oxford University Press.

[ref41] Paganos P. Voronov D. Musser J. M. Arendt D. Arnone M. I. (2021). Single-cell RNA sequencing of the *Strongylocentrotus purpuratus* larva reveals the blueprint of major cell types and nervous system of a non-chordate deuterostome. eLife 10:e70416. doi: 10.7554/eLife.70416 34821556 PMC8683087

[ref42] Parks A. L. Parr B. A. Chin J.-E. Leaf D. S. Raff R. A. (1988). Molecular analysis of heterochronic changes in the evolution of direct developing sea urchins. J. Evol. Biol. 1, 27–44. doi: 10.1046/j.1420-9101.1988.1010027.x

[ref43] Pearse J. S. (1969). Slow developing demersal embryos and larvae of the Antarctic Sea star *Odontaster validus*. Mar. Biol. 3, 110–116. doi: 10.1007/BF00353429

[ref44] Perillo M. Paganos P. Mattiello T. Cocurullo M. Oliveri P. Arnone M. I. (2018). New neuronal subtypes with a “pre-pancreatic” signature in the sea urchin *Stongylocentrotus purpuratus*. Front. Endocrinol. 9:650. doi: 10.3389/fendo.2018.00650, PMID: 30450080 PMC6224346

[ref45] Pires A. Woollacott R. M. (1997). Serotonin and dopamine have opposite effects on phototaxis in larvae of the bryozoan *Bugula neritina*. Biol. Bull. 192, 399–409. doi: 10.2307/1542749, PMID: 28581842

[ref46] Ryberg E. (1974). The localization of biogenic amines in the Echinopluteus. Acta Zool. 55, 179–189. doi: 10.1111/j.1463-6395.1974.tb00193.x

[ref47] Sakano D. Choi S. Kataoka M. Shiraki N. Uesugi M. Kume K.. (2016). Dopamine D2 receptor-mediated regulation of pancreatic β cell mass. Stem Cell Rep. 7, 95–109. doi: 10.1016/j.stemcr.2016.05.015, PMID: 27373926 PMC4944721

[ref48] Scheltema R. S. (1977). Dispersal of marine invertebrate organisms: paleogeographic and biostratigraphic implications. Concepts Methods Biostratigr. 1, 73–108.

[ref49] Schindelin J. Arganda-Carreras I. Frise E. Kaynig V. Longair M. Pietzsch T.. (2012). Fiji: an open-source platform for biological-image analysis. Nat. Methods 9, 676–682. doi: 10.1038/nmeth.2019, PMID: 22743772 PMC3855844

[ref50] Shiba K. Mogami Y. Baba S. A. (2002). Ciliary movement of sea-urchin embryos. Nat. Sci. Rep. Ochanomizu. Univ. 53, 49–54.

[ref51] Slota L. A. McClay D. R. (2018). Identification of neural transcription factors required for the differentiation of three neuronal subtypes in the sea urchin embryo. Dev. Biol. 435, 138–149. doi: 10.1016/j.ydbio.2017.12.015, PMID: 29331498 PMC5837949

[ref52] Smith K. K. (2003). Time’s arrow: heterochrony and the evolution of development. Int. J. Dev. Biol. 47, 613–621. PMID: 14756337

[ref53] Soliman S. (1983). Pharmacological control of ciliary activity in the young sea urchin larva. Effects of monoaminergic agents. Comp. Biochem. Physiol. C Comp. Pharmacol. Toxicol. 76, 181–191. doi: 10.1016/0742-8413(83)90061-0 6139247

[ref54] Strathmann R. R. Fenaux L. Strathmann M. F. (1992). Heterochronic developmental plasticity in larval sea urchins and its implications for evolution of nonfeeding larvae. Evolution 46, 972–986. doi: 10.1111/j.1558-5646.1992.tb00613.x, PMID: 28564401

[ref55] Thorson G. (1961). “Length of pelagic larval life in marine bottom invertebrates as related to larval transport by ocean currents” in Oceanography. ed. Sears M. (Washington, DC: American Association for the Advancement of Science)

[ref56] Uhler G. C. Huminski P. T. Les F. T. Fong P. P. (2000). Cilia-driven rotational behavior in gastropod (*Physa elliptica*) embryos induced by serotonin and putative serotonin reuptake inhibitors (SSRIs). J. Exp. Zool. 286, 414–421. doi: 10.1002/(SICI)1097-010X(20000301)286:4<414::AID-JEZ9>3.0.CO;2-B, PMID: 10684564

[ref57] Voronezhskaya E. E. Hiripi L. Elekes K. Croll R. P. (1999). Development of catecholaminergic neurons in the pond snail, *Lymnaea stagnalis*: I. Embryonic development of dopamine-containing neurons and dopamine-dependent behaviors. J. Comp. Neurol. 404, 285–296. doi: 10.1002/(SICI)1096-9861(19990215)404:3<285::AID-CNE1>3.0.CO;2-S, PMID: 9952348

[ref58] Wada Y. Mogami Y. Baba S. A. (1997). Modification of ciliary beating in sea urchin larvae induced by neurotransmitters: beat-plane rotation and control of frequency fluctuation. J. Exp. Biol. 200, 9–18. doi: 10.1242/jeb.200.1.9, PMID: 9317232

[ref59] Walentek P. Bogusch S. Thumberger T. Vick P. Dubaissi E. Beyer T.. (2014). A novel serotonin-secreting cell type regulates ciliary motility in the mucociliary epidermis of *Xenopus* tadpoles. Development 141, 1526–1533. doi: 10.1242/dev.102343, PMID: 24598162

[ref60] Yaguchi S. Katow H. (2003). Expression of tryptophan 5-hydroxylase gene during sea urchin neurogenesis and role of serotonergic nervous system in larval behavior. J. Comp. Neurol. 466, 219–229. doi: 10.1002/cne.10865, PMID: 14528449

[ref61] Yaguchi S. Taniguchi Y. Suzuki H. Kamata M. Yaguchi J. (2022). Planktonic Sea urchin larvae change their swimming direction in response to strong photoirradiation. PLoS Genet. 18:e1010033. doi: 10.1371/journal.pgen.1010033, PMID: 35143488 PMC8830728

[ref62] Yaroslavtseva L. M. Sergeeva E. P. Kashenko S. D. (2002). The vertical distribution of larvae of the sea urchin *Strongylocentrotus intermedius* in superficial desalination conditions. Russ. J. Mar. Biol. 28, 191–196. doi: 10.1023/A:1016849404377

[ref63] Zhang X. Jia S. Chen Z. Chong Y. L. Xie H. Feng D.. (2018). Cilia-driven cerebrospinal fluid flow directs expression of urotensin neuropeptides to straighten the vertebrate body axis. Nat. Genet. 50, 1666–1673. doi: 10.1038/s41588-018-0260-3, PMID: 30420648

